# Supplementary material for: Activating lattice oxygen in NiFe-based (oxy)hydroxide for water electrolysis
Source: Nat Commun. 2022 Apr 21;13:2191. doi: 10.1038/s41467-022-29875-4 (PMC9023528; doi:10.1038/s41467-022-29875-4)
Supplement: Supplementary file 1 — Supplementary Information [file 41467_2022_29875_MOESM1_ESM.pdf]

Supplementary Information

Activating lattice oxygen in NiFe-based (oxy)hydroxide for water electrolysis

Zuyun He[1,5], Jun Zhang[2,5], Zhiheng Gong[1], Hang Lei[3], Deng Zhou[4], Nian Zhang[4], Wenjie Mai[3], Shijun Zhao[2*], Yan Chen[1*]

[1]School of Environment and Energy, State Key Laboratory of Pulp and Paper Engineering, South China University of Technology, Guangzhou, Guangdong, 510006, China

[2]Department of Mechanical Engineering, City University of Hong Kong, Hong Kong, China

[3]Siyuan Laboratory, Guangdong Provincial Engineering Technology Research Centre of Vacuum Coating Technologies and New Energy Materials, Department of Physics, Jinan University, Guangzhou, Guangdong, 510632, China

[4]State Key Laboratory of Functional Materials for Informatics, Shanghai Institute of Microsystem and Information Technology, Chinese Academy of Sciences, Shanghai, 200050, China

[5]These authors contributed equally: Zuyun He, Jun Zhang

*Corresponding authors:

E-mail addresses: escheny@scut.edu.cn (Yan Chen), shijzhao@cityu.edu.hk (Shijun Zhao)

Supplementary Figure

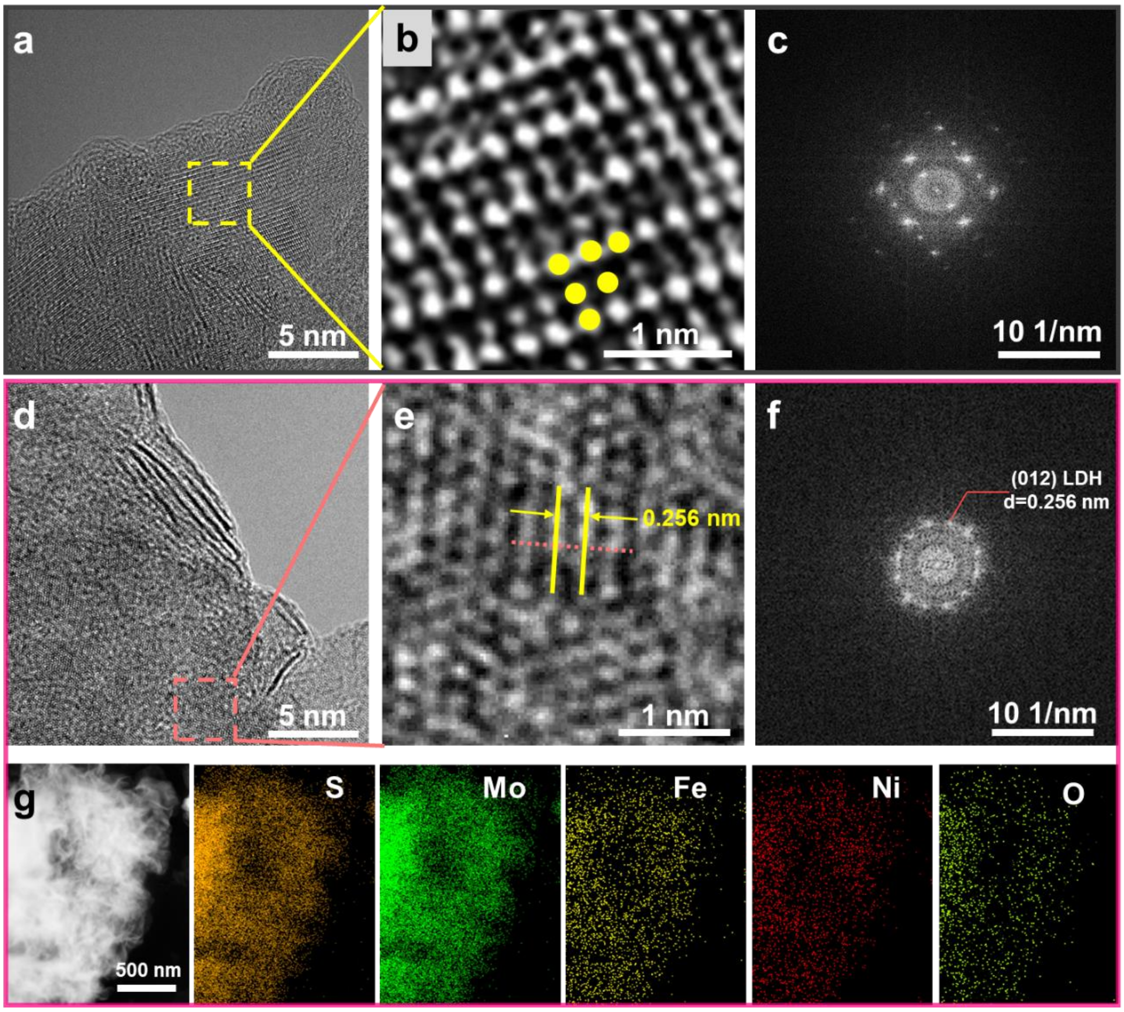

Supplementary Fig. 1. Structure characterizations of the 1T-MoS_2 nanosheets and MoS_2/NiFe LDH pre-catalysts. High-resolution transmission electron microscopy (HRTEM) images with **a** low magnification and **b** high magnification and **c** the corresponding Fast Fourier transform pattern for the 1T-MoS_2 nanosheets. HRTEM images with **d** low magnification and **e** high magnification and **f** the corresponding Fast Fourier transform pattern for the MoS_2/NiFe LDH pre-catalysts. **g** HRTEM image and energy dispersive spectroscopy (EDS) mapping for the MoS_2/NiFe LDH pre-catalysts .

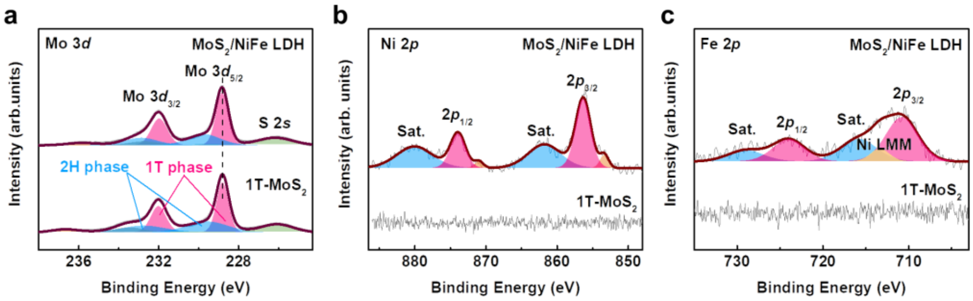

Supplementary Fig. 2. XPS spectra of cations in the 1T-MoS_2 nanosheets and MoS_2/NiFe LDH pre-catalysts. a Mo 3*d*, **b** Ni 2*p*, and **c** Fe 2*p* XPS spectra.

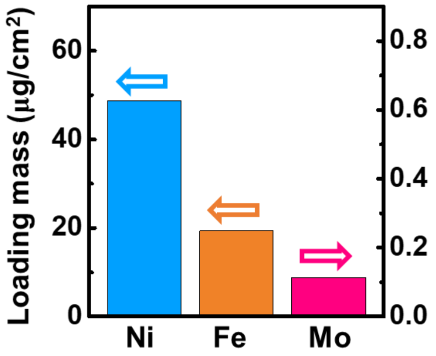

Supplementary Fig. 3. Chemical composition characterizations for MoNiFe-27% (oxy)hydroxide. The content of Ni, Fe, Mo cation in MoNiFe-27% (oxy)hydroxide determined by ICP-OES measurment.

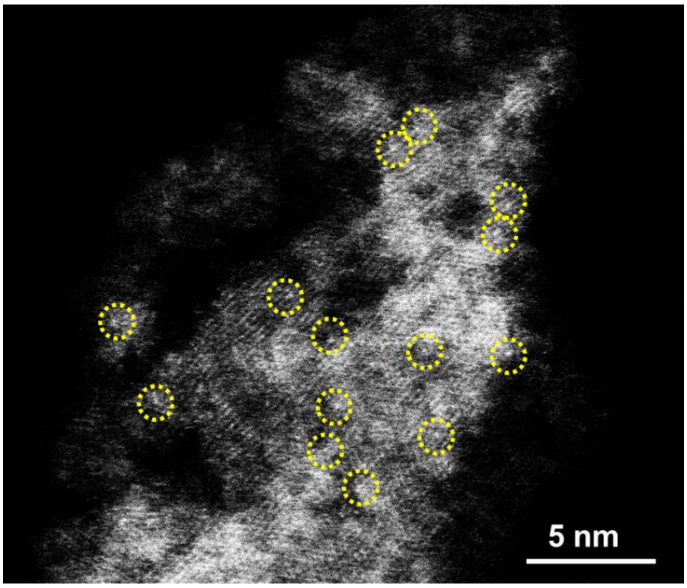

Supplementary Fig. 4. Mo dopant identification. The HAADF-STEM image of MoNiFe (oxy)hydroxide.

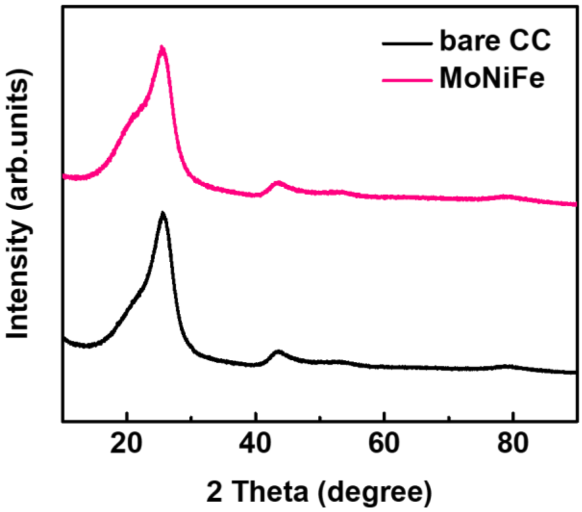

Supplementary Fig. 5. Crystal structure characterizations. The X-ray diffraction pattern of bare carbon cloths and MoNiFe (oxy)hydroxide.

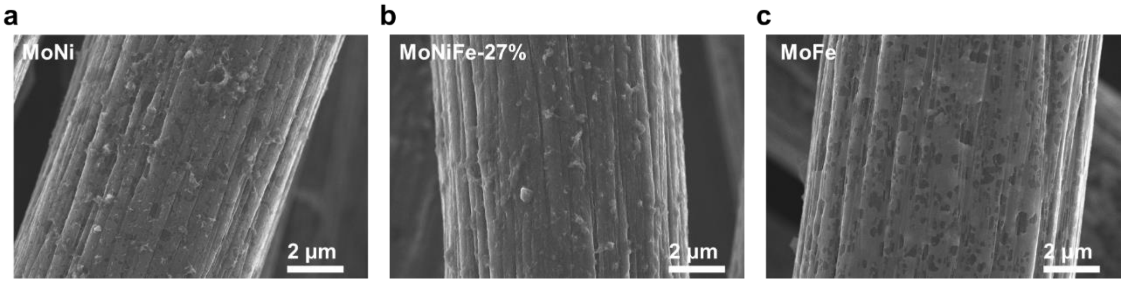

Supplementary Fig. 6. Morphology characterizations of MoNiFe (oxy)hydroxide.

SEM images of MoNiFe (oxy)hydroxide with various Fe contents of **a** 0%, **b** 27%, **c** 100% .

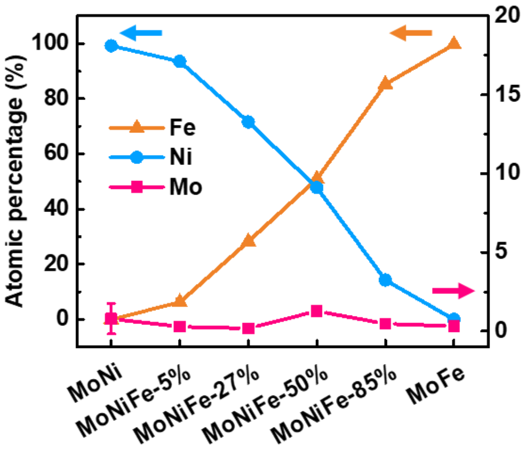

Supplementary Fig. 7. Chemical composition characterizations for MoNiFe (oxy)hydroxide. The metal contents in MoNiFe-x% (oxy)hydroxide, (x=0%, 5%, 27%, 50%, 85%, 100%) determined by ICP-OES measurement. The error bar represents the standard deviation of results obtained from multiple samples.

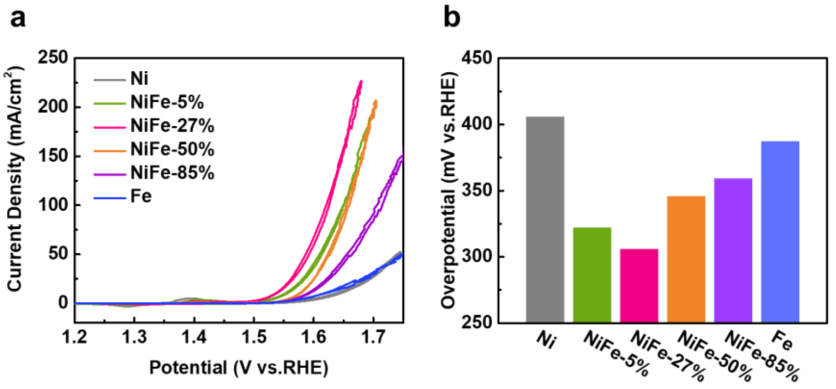

Supplementary Fig. 8. The OER activities of NiFe (oxy)hydroxide with various Fe content. a Cyclic voltammetry polarization curves. **b** Overpotential comparison for the NiFe (oxy)hydroxide with Fe content of 0%, 5%, 27%, 50%, 85% and 100% at the current density of 10 mA/cm^2.

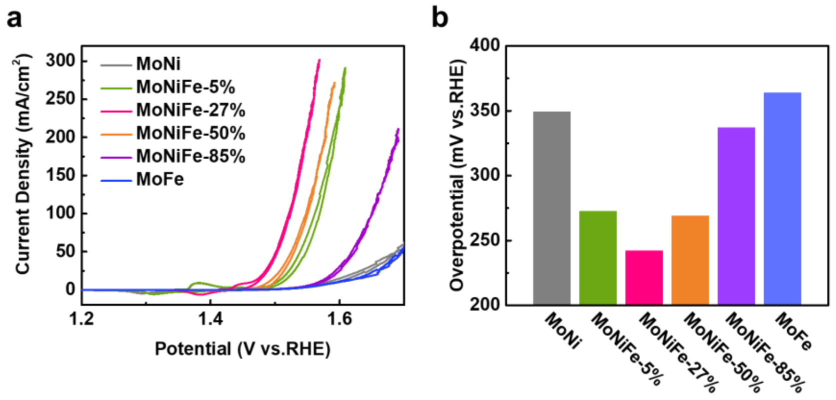

Supplementary Fig. 9. The OER activities of MoNiFe (oxy)hydroxide with various Fe content. a Cyclic voltammetry polarization curves. **b** Overpotential comparison for the MoNiFe (oxy)hydroxide with various Fe content of 0%, 5%, 27%, 50%, 85% and 100% at the current density of 10 mA/cm^2.

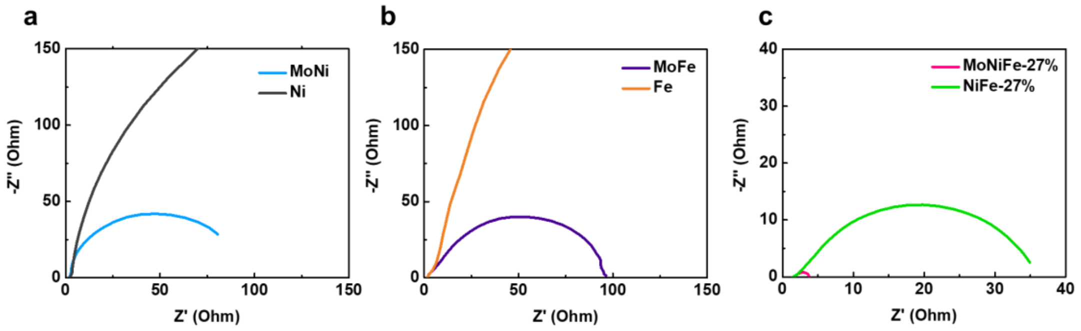

Supplementary Fig. 10. Comparision of electrochemical impedance spectroscopies. a Ni and MoNi, **b** Fe and MoFe, **c** NiFe-27% and MoNiFe-27% (oxy)hydroxide.

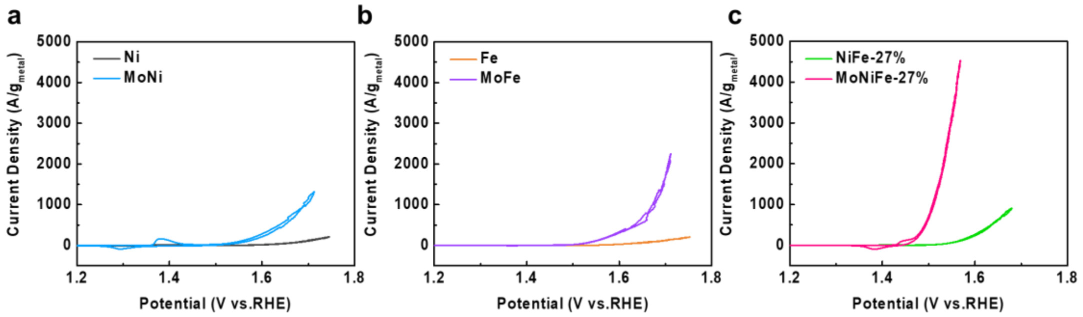

Supplementary Fig. 11. Cyclic voltammetry polarization curves normalized by metal mass of catalysts. a Ni and MoNi, **b** Fe and MoFe, **c** NiFe-27% and MoNiFe-27% (oxy)hydroxide.

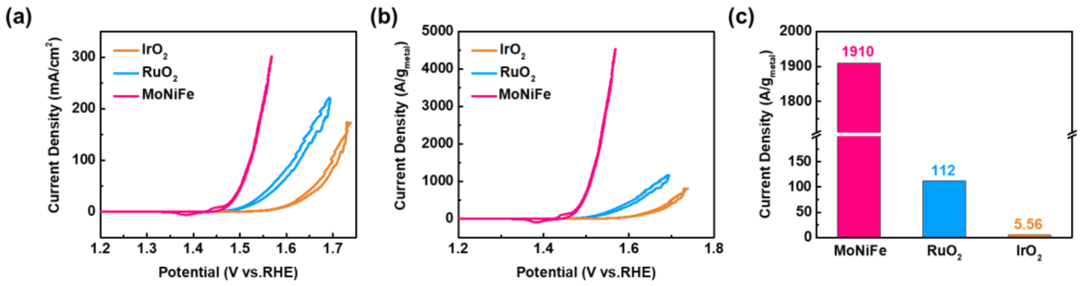

Supplementary Fig. 12. The OER activities of MoNiFe (oxy)hydroxide, RuO_2 and IrO_2. Cyclic voltammetry polarization curves normalized by **a** geometric area and **b** loading mass. **c**, The specific mass activity at the overpotential of 300 mV.

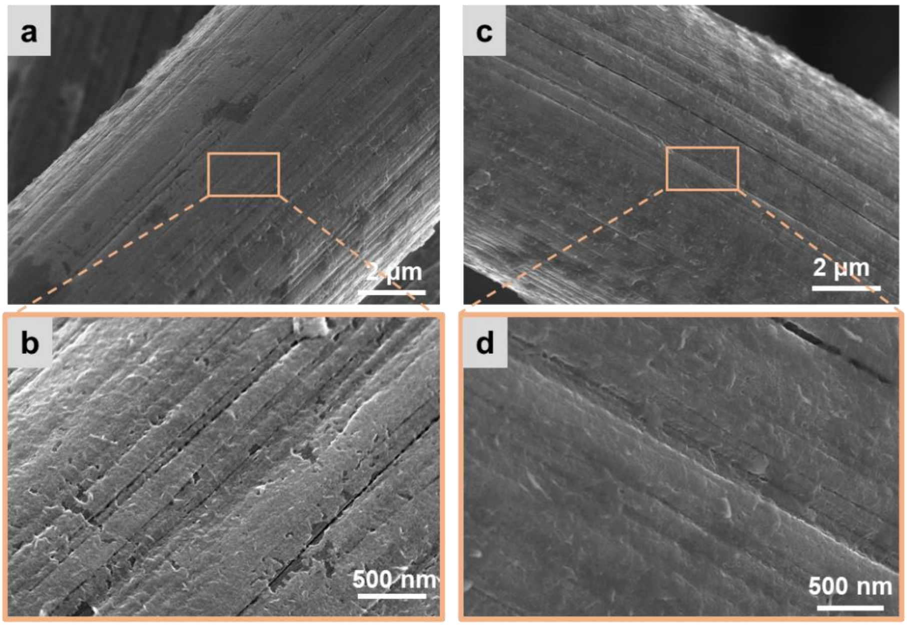

Supplementary Fig. 13. Morphology characterizations. The SEM images of the MoNiFe (oxy)hydroxide **a-b** before and **c-d** after CP measurement at 100 mA/cm^2 for 65 h.

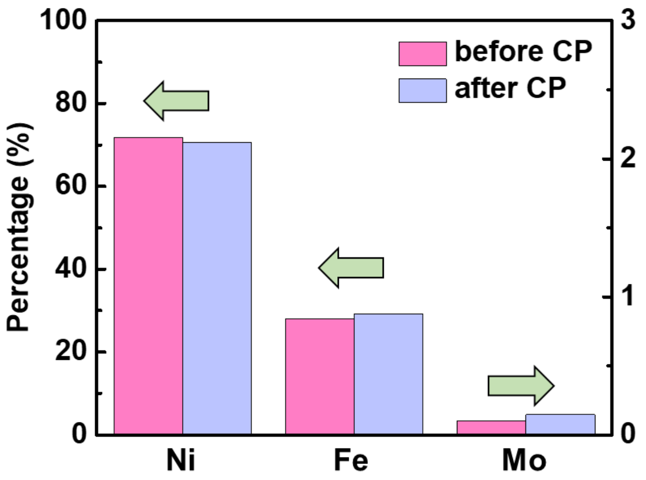

Supplementary Fig. 14. Chemical composition characterizations. The comparison of Ni, Fe, Mo content in MoNiFe (oxy)hydroxide before and after CP measurement determined by ICP-OES measurment.

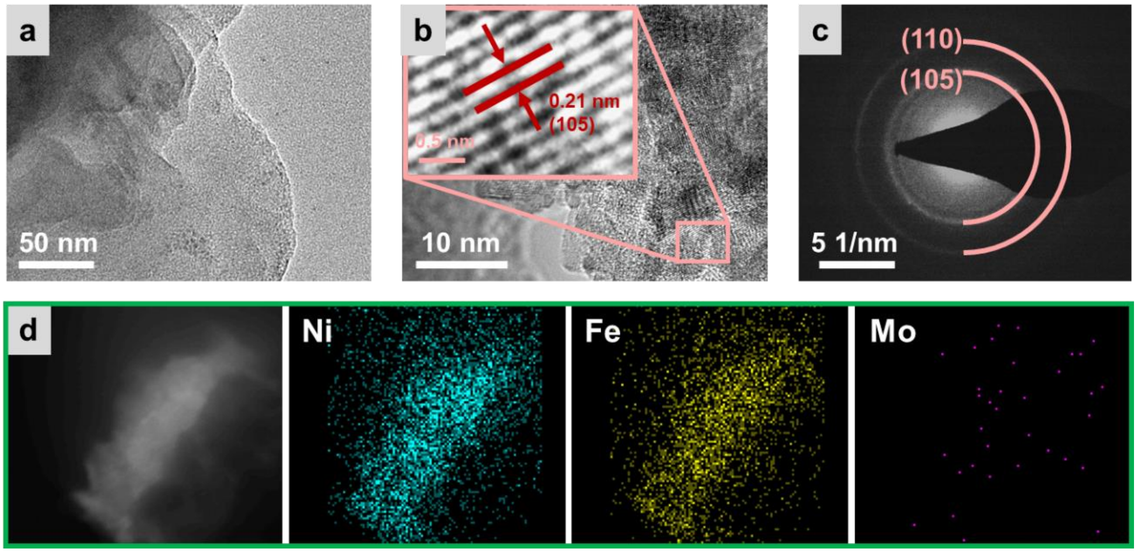

Supplementary Fig. 15. Structure characterizations of MoNiFe (oxy)hydroxide after CP measurement at 100 mA/cm^2 for 65 h. High resolution transmission electron microscopy (HRTEM) images with **a** low magnification and **b** high magnification. **c** The corresponding selected area electron diffraction (SAED) pattern. **d** Energy dispersive spectroscopy (EDS) mapping of Ni, Fe, and Mo elements.

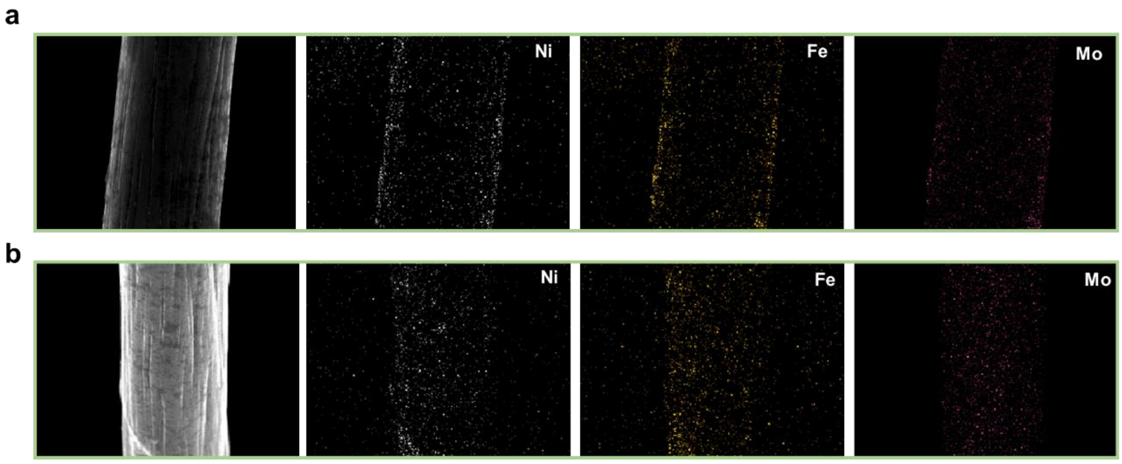

Supplementary Fig. 16. Element distribution characterizations. The SEM-EDS mapping of the MoNiFe (oxy)hydroxide **a** before and **b** after CP measurement at 100 mA/cm^2 for 65 h.

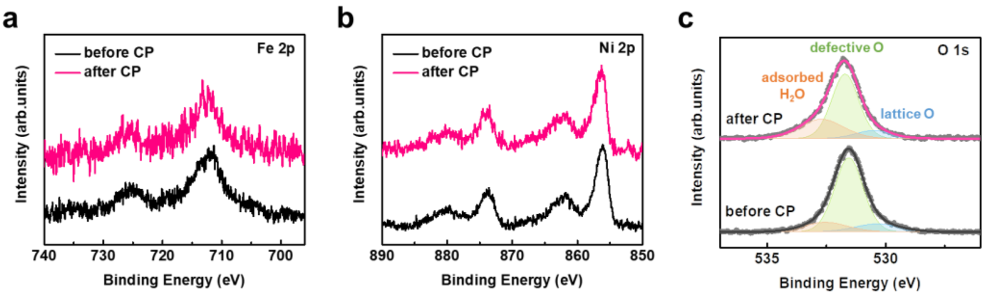

Supplementary Fig. 17. XPS spectra of MoNiFe (oxy)hydroxide before and after CP measurement. a The Fe 2*p*, **b** Ni 2*p*, and **c** O 1*s* XPS spectra.

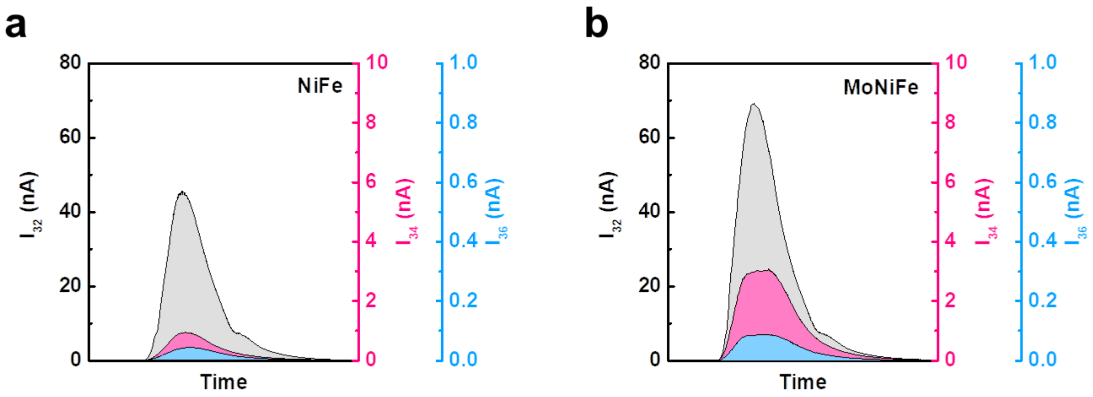

Supplementary Fig. 18. The differential electrochemical mass spectrometry (DEMS) signals of $^{16}O_2$ (I_{32}), $^{16}O^{18}O$ (I_{34}), and $^{18}O_2$ (I_{36}). a NiFe (oxy)hydroxide, **b** MoNiFe (oxy)hydroxide.

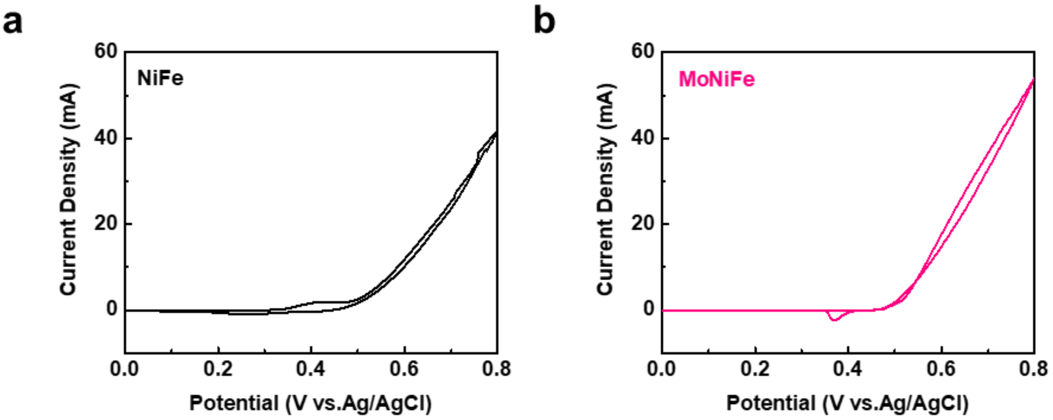

Supplementary Fig. 19. The cyclic voltammograms curves during DEMS measurement. a NiFe (oxy)hydroxide, **b** MoNiFe (oxy)hydroxide.

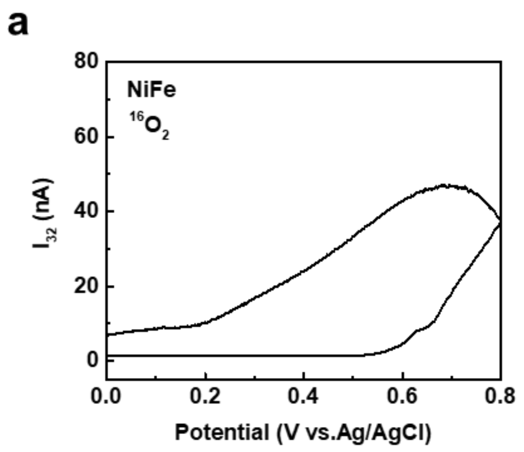

Supplementary Fig. 20. The DEMS signal of m/z=32 ($^{16}O_2$) as a function of applied potential during OER measurement. a NiFe (oxy)hydroxide, **b** MoNiFe (oxy)hydroxide.

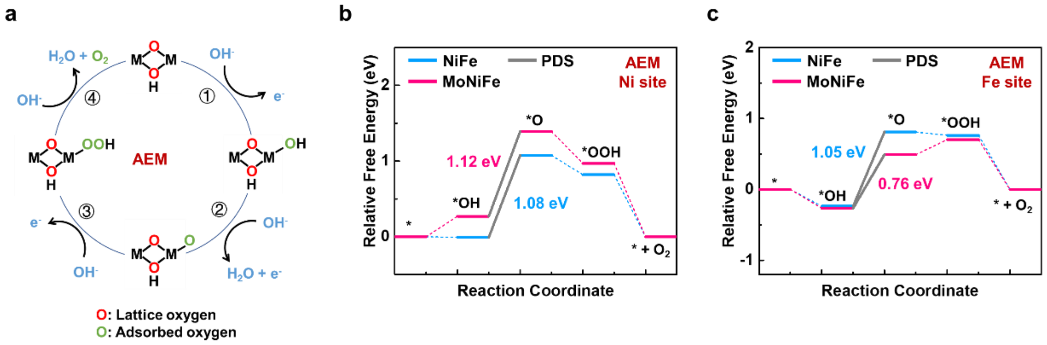

Supplementary Fig. 21. AEM pathway calcualtions. a Schematic illustration of the AEM pathway. The Gibbs free energy diagrams of OER in AEM pathway on **b** Ni site and **c** Fe site on NiFe and MoNiFe (oxy)hydroxide.

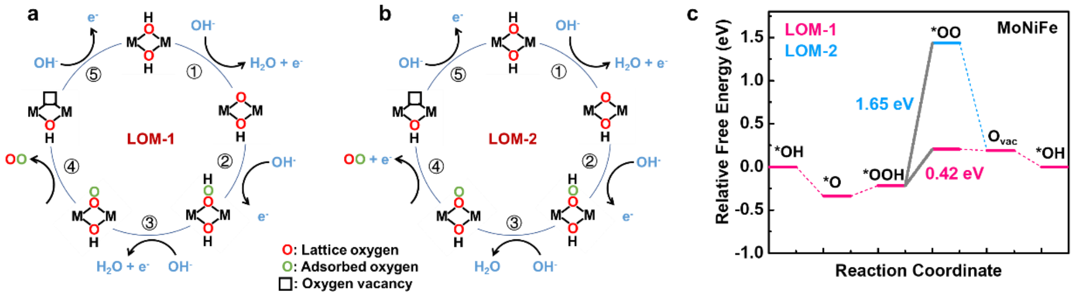

Supplementary Fig. 22. Two possible LOM pathways on MoNiFe (oxy)hydroxide. Schematic illustration of **a** LOM-1 and **b** LOM-2. **c** The Gibbs free energy diagrams of OER in LOM-1 and LOM-2 pathways on MoNiFe (oxy)hydroxide.

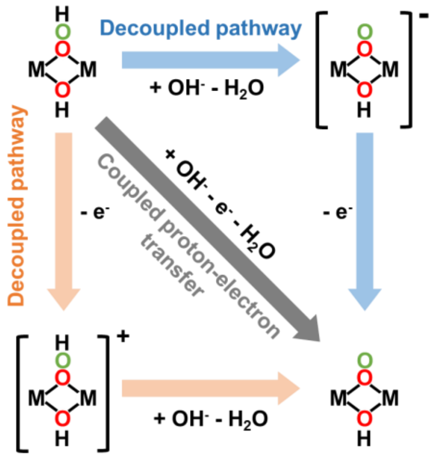

Supplementary Fig. 23. Non-concerned proton-electron transfer process. Illustration of decoupled proton-electron transfer of the potential determining step in MoNiFe (oxy)hydroxide.

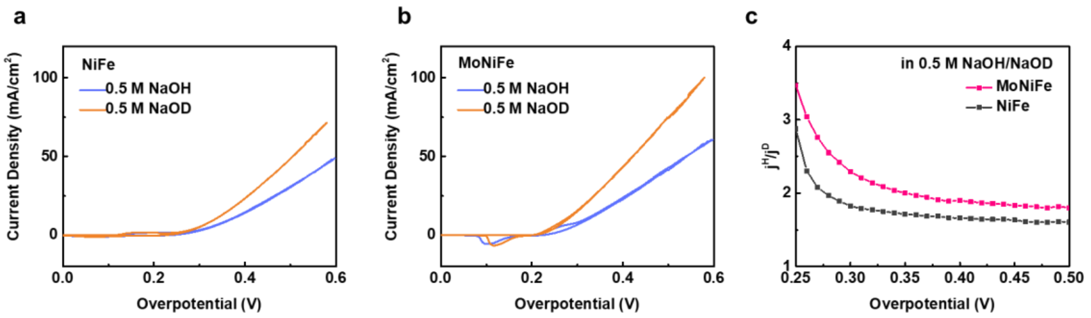

Supplementary Fig. 24. The dependence of OER activity on proton activity. The LSV curves for **a** NiFe and **b** MoNiFe (oxy)hydroxide measured in 0.5 M NaOH and 0.5 M NaOD solution. The LSV curves are without iR compensation. **c** The kinetic isotope effect of MoNiFe and NiFe (oxy)hydroxide in 0.5 M NaOH/NaOD.

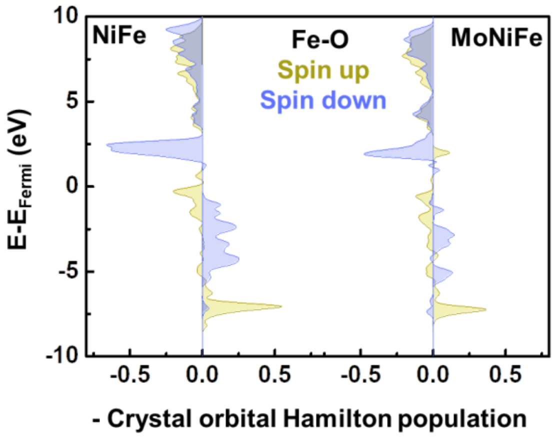

Supplementary Fig. 25. Crystal orbital Hamilton populations calcualtions.

Crystal orbital Hamilton populations of the Fe-O bond in NiFe and MoNiFe (oxy)hydroxide.

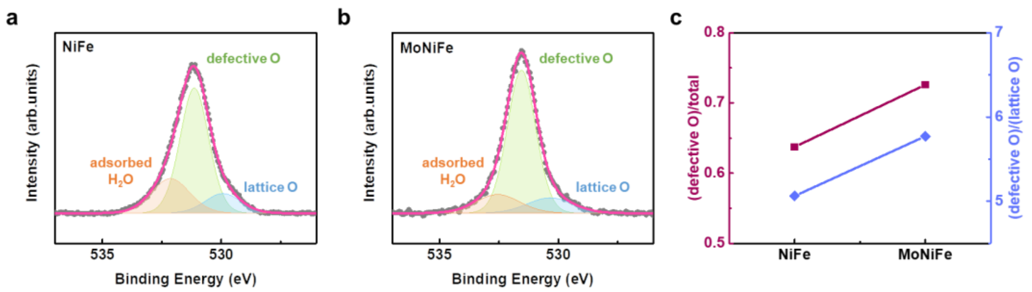

Supplementary Fig. 26. Oxygen vacancy analysis. The O 1*s* XPS spectrum of **a** NiFe and **b** MoNiFe (oxy)hydroxide. **c** Comparison of fitting results for (defective O)/total and (defective O)/(lattice O) ratio.

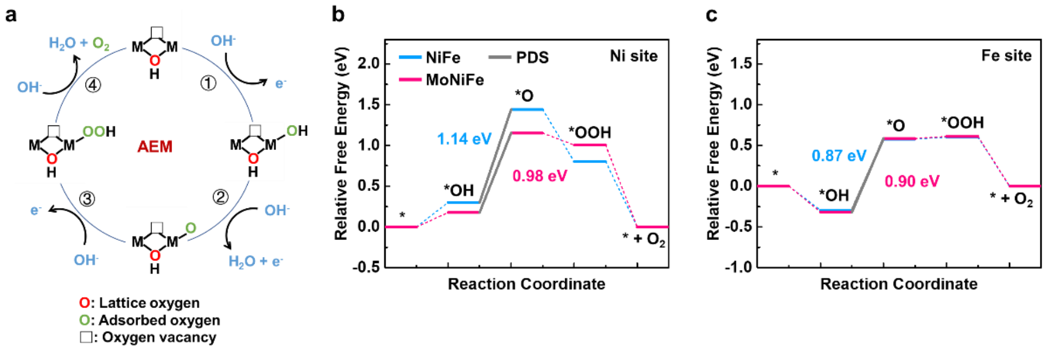

Supplementary Fig. 27. AEM pathway calculations for NiFe and MoNiFe (oxy)hydroxide with oxygen vacancy. a Schematic illustration of the AEM pathway. The Gibbs free energy diagrams of OER in the AEM pathway on **b** Ni site and **c** Fe site in NiFe and MoNiFe (oxy)hydroxide with oxygen vacancy.

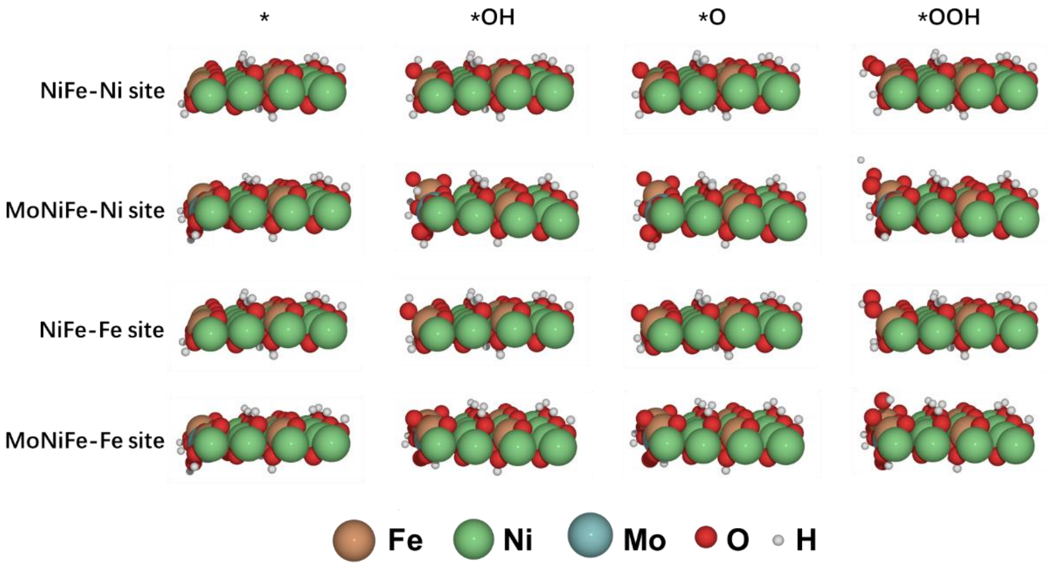

Supplementary Fig. 28. The configurations of AEM pathway calculation. The adsorption configuration of reaction intermediate on NiFe and MoNiFe (oxy)hydroxide with oxygen vacancy in the AEM mechanism.

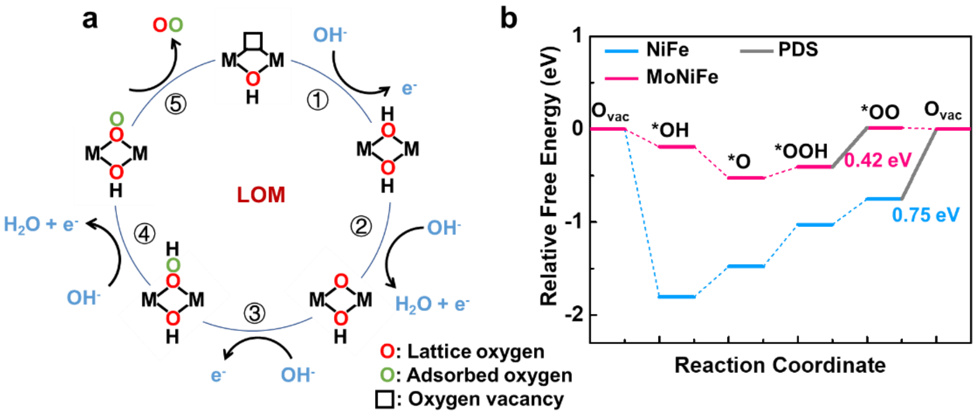

Supplementary Fig. 29. LOM pathway calculations for NiFe and MoNiFe (oxy)hydroxide with oxygen vacancy. a Schematic illustration of the LOM pathway. **b** The Gibbs free energy diagrams of OER in the LOM pathway on NiFe and MoNiFe (oxy)hydroxide with oxygen vacancy.

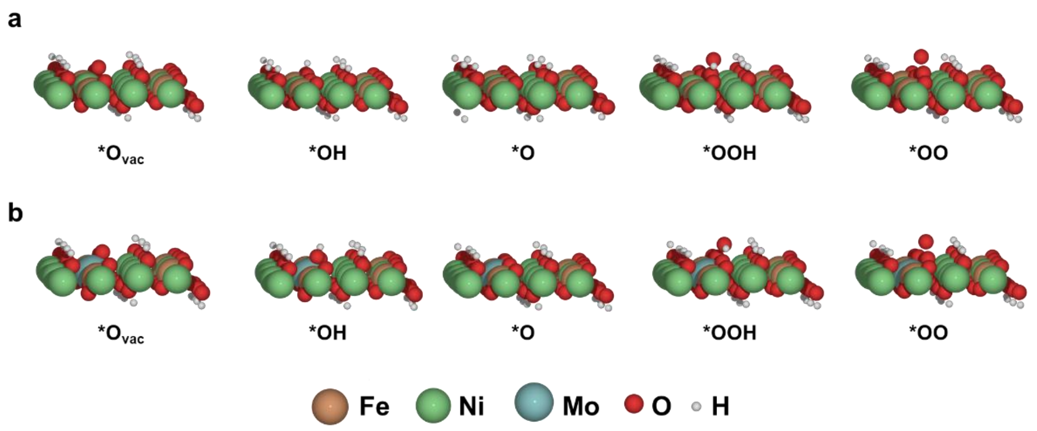

Supplementary Fig. 30. The configurations of LOM pathway calculation. The adsorption configuration of reaction intermediate on NiFe and MoNiFe (oxy)hydroxide with oxygen vacancy in the LOM mechanism.

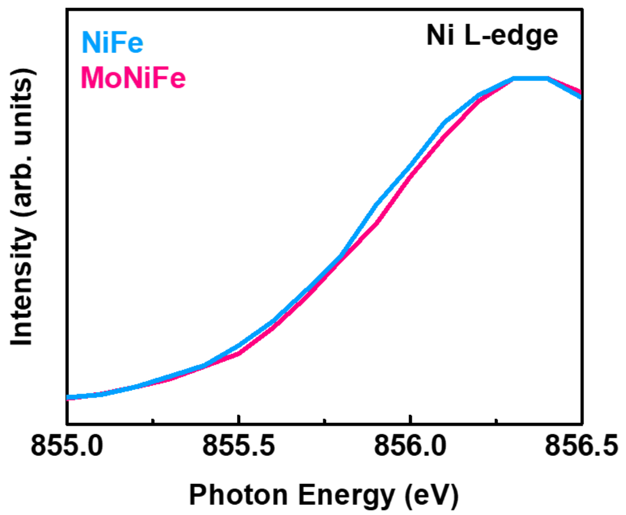

Supplementary Fig. 31. Ni L-edge sXAS spectra comparison. The intensity-normalized Ni L-edge sXAS spectra of NiFe and MoNiFe (oxy)hydroxide.

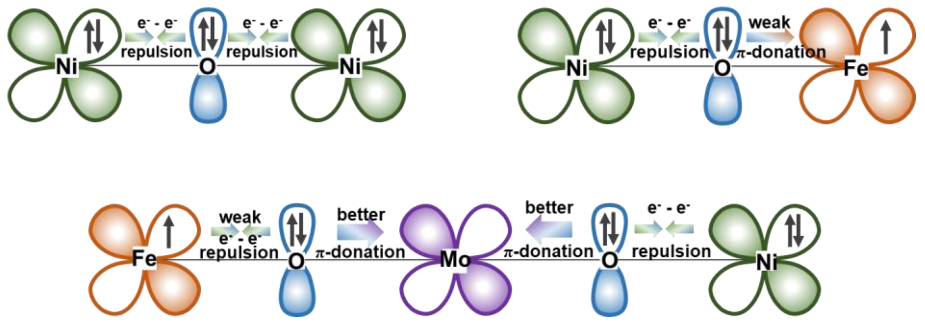

Supplementary Fig. 32. **Electron transfer analysis.** Schematic illustration of the electron transfer among Ni, Fe, Mo, O in Ni, NiFe and MoNiFe (oxy)hydroxide.

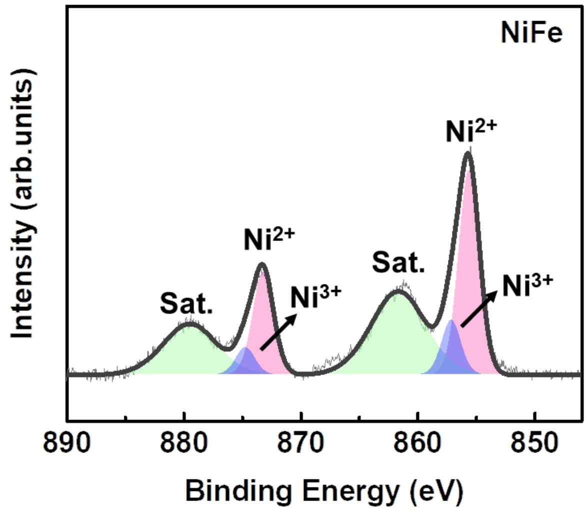

Supplementary Fig. 33. **Ni valence state characterization.** Ni 2*p* XPS spectra of NiFe (oxy)hydroxide.

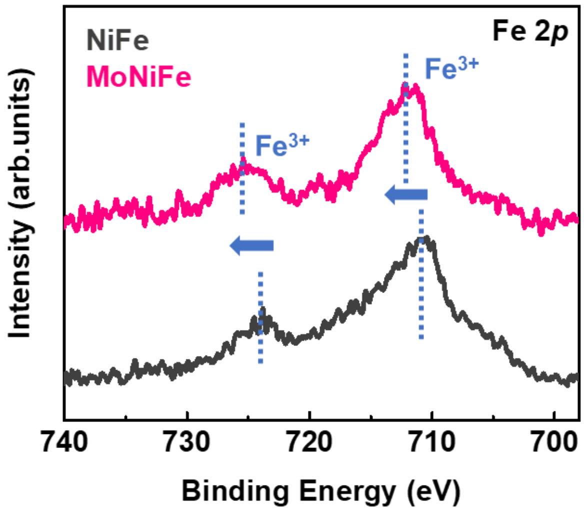

Supplementary Fig. 34. **Fe valence state characterization.** The Fe 2*p* XPS spectra of NiFe and MoNiFe (oxy)hydroxide.

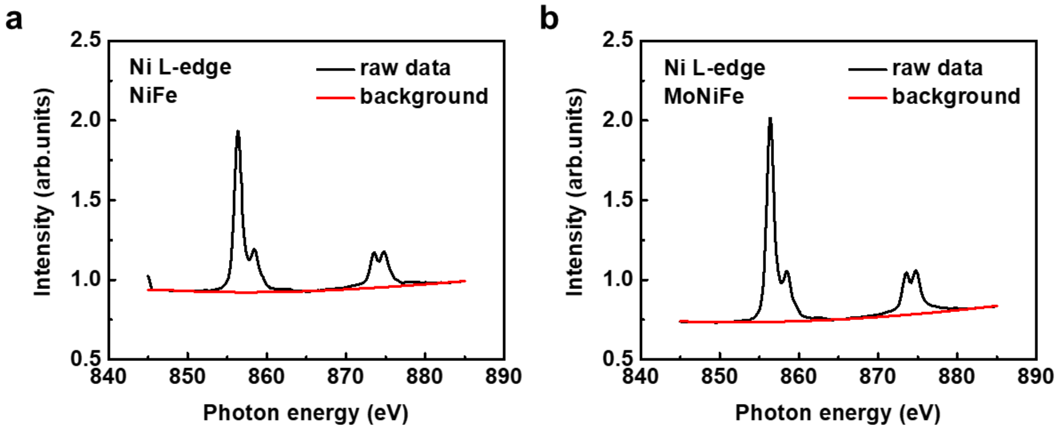

Supplementary Fig. 35. The raw data of the Ni L-edge XAS sepctra. a NiFe (oxy)hydroxide, **b** MoNiFe (oxy)hydroxide. The red lines represent the background.

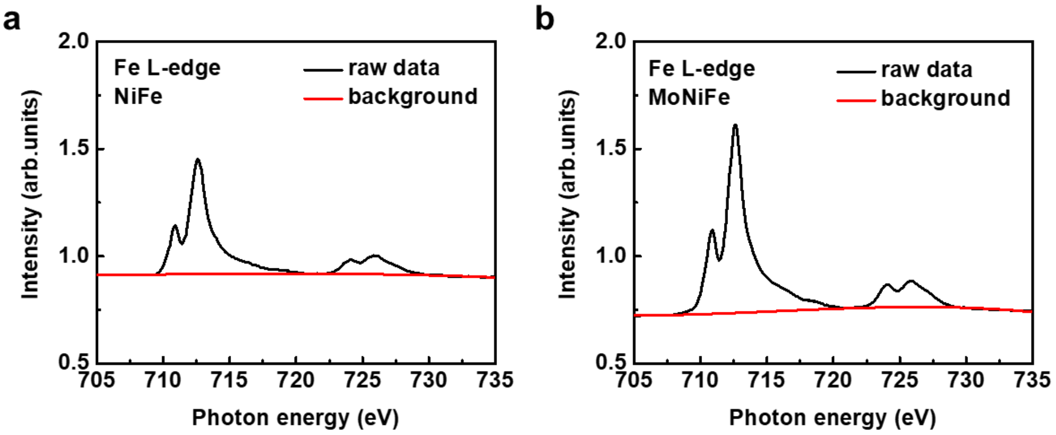

Supplementary Fig. 36. The raw data of the Fe L-edge XAS spectra. a NiFe (oxy)hydroxide, **b** MoNiFe (oxy)hydroxide. The red lines represent the background.

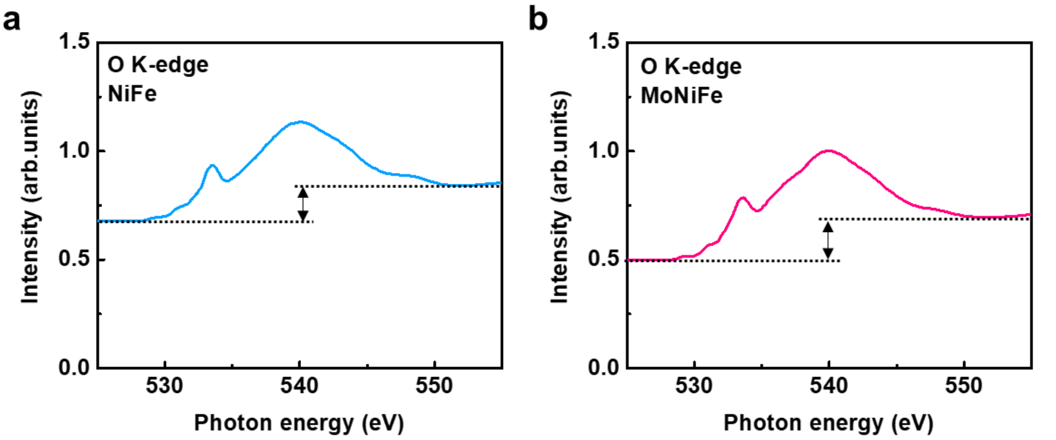

Supplementary Fig. 37. The raw data of the O K-edge XAS spectra. a NiFe (oxy)hydroxide, **b** MoNiFe (oxy)hydroxide. The dash lines represent the edge jump at O K-edge.

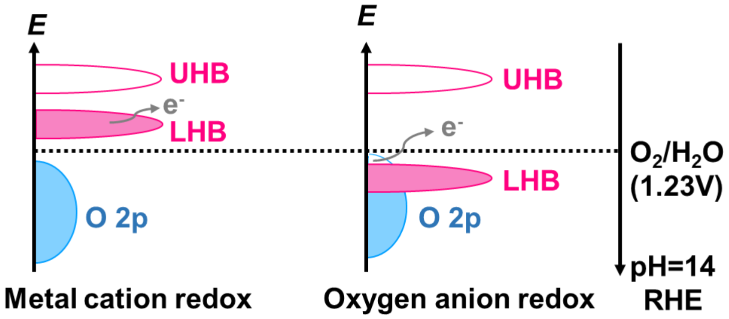

Supplementary Fig. 38. **Competition between metal cation redox and oxygen anion redox.** The schematic representation of cation/anion redox chemistry determined by *d*-*d* Coulomb interaction (U) and O 2*p* band center position relative to Fermi level.

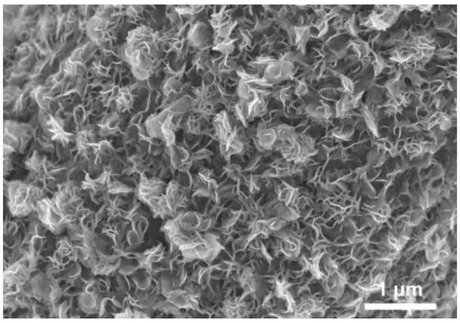

Supplementary Fig. 39. **Morphology characterizations.** SEM image of the MoS_2/NiFe LDH cathode after HER.

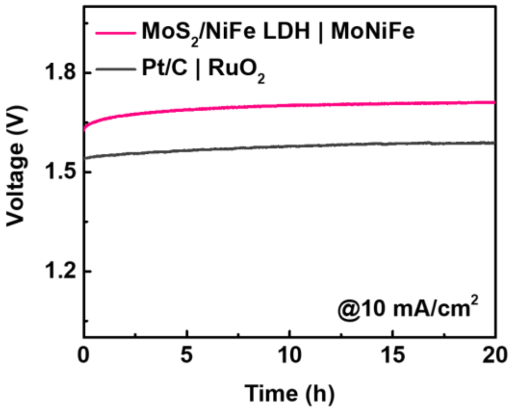

Supplementary Fig. 40. **Overall water sppliting stability measurement.**

Chronopotentiometry curves at the current density of 10 mA/cm^2 of the electrolytic cell with MoS_2/NiFe LDH|MoNiFe coupled electrodes and the reference cell with Pt/C|RuO_2 coupled electrodes.

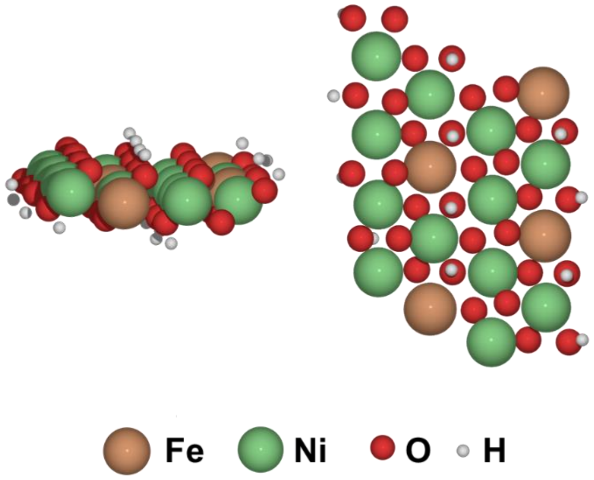

Supplementary Fig. 41. Slab model for DFT caculations. The slab model of NiFe (oxy)hydroxide.

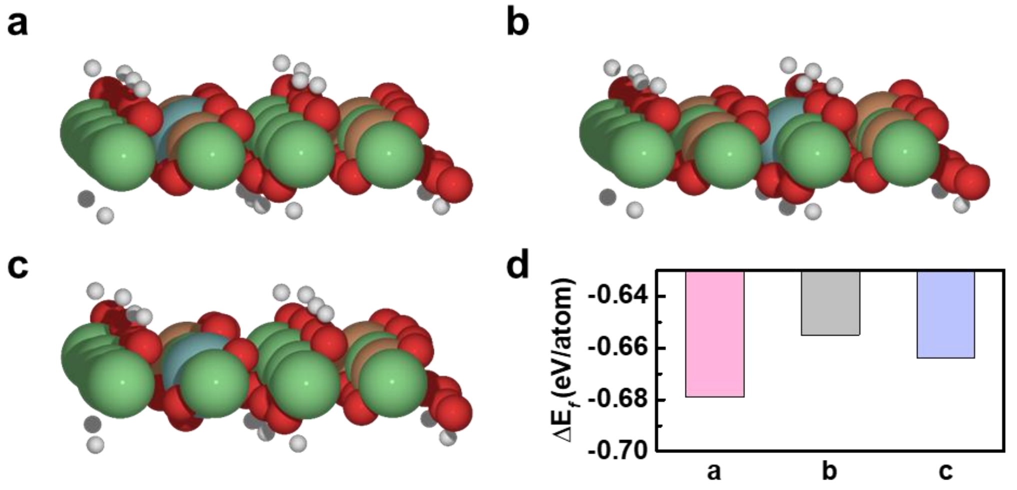

Supplementary Fig. 42. Doping (Ni,Fe)(OH)2 with Mo at different sites. a, Mo atom replaces the surface Ni and exposes to the vacuum. **b**, Mo atom replaces surface Ni atom without the exposure to vacuum. **c**, Mo atom replaces the surface Fe atom. **d**, The comparison of the formation energy (ΔE_f) of configuration a-c.

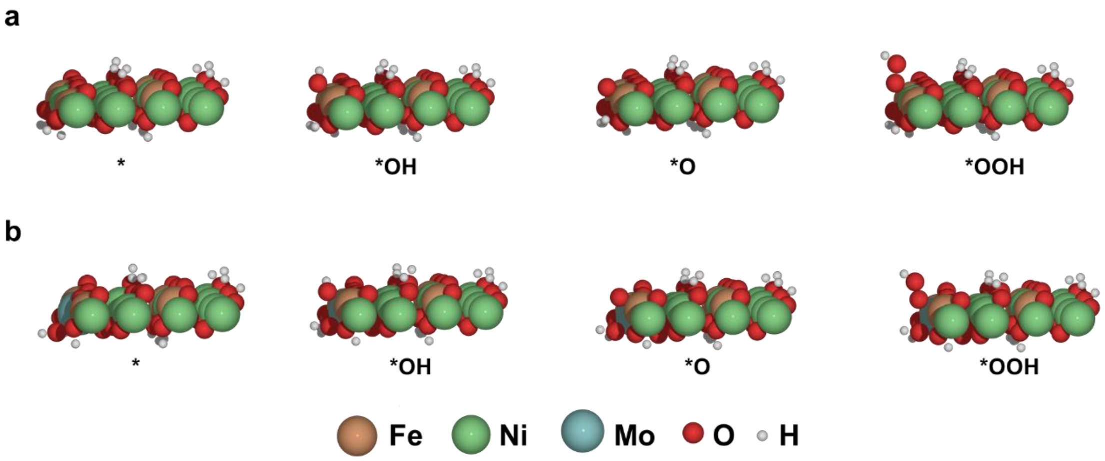

Supplementary Fig. 43. **Configurations for AEM pathway calcuations.** The configurations of NiFe (a) and MoNiFe (b) (oxy)hydroxide in AEM mechanism.

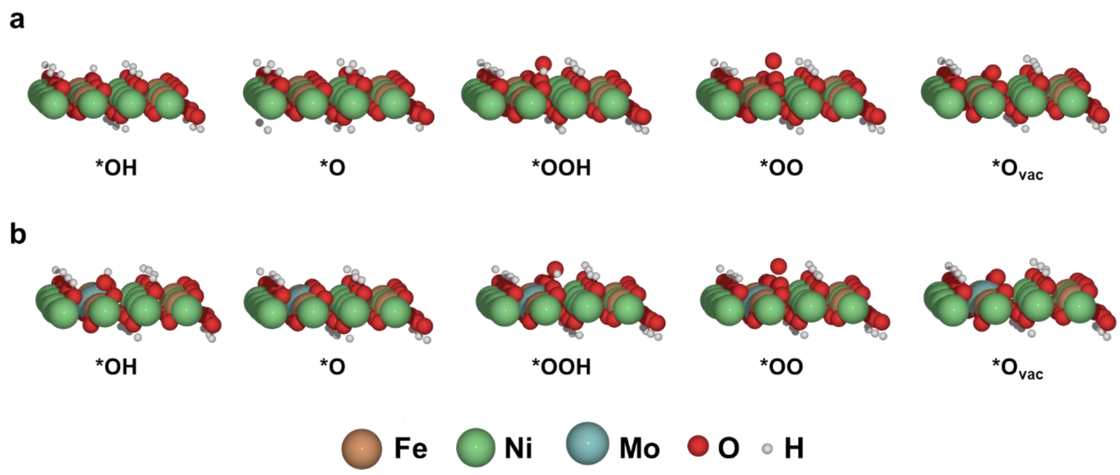

Supplementary Fig. 44. **Configurations for LOM pathway calcuations.** The configurations of NiFe (a) and MoNiFe (b) (oxy)hydroxide in LOM mechanism.

Supplementary Table 1. The comparison of overall water splitting performance.

The applied voltage at the current density of 100 mA/cm^2 for MoS_2/NiFe LDH|MoNiFe and other noble-metal-free electrocatalysts in recent reported literatures.

Catalyst	Voltage (V) (100 mA/cm^2)	Reference
MoNiFe (oxy)hydroxide	1.728	This work
CuNi@NiFeCu/CP	1.8	Ref [1]
LSC/K-$MoSe_2$	1.95	Ref [2]
CoFe-NA2/NF	1.95	Ref [3]
MnCo-CH@NiFe-OH	1.69	Ref [4]
NiFe-LDH/$Ni(OH)_2$	1.81	Ref [5]
2-APSC/NF	1.72	Ref [6]
Co/CoO@NC@CC	1.75	Ref [7]
1T-Fe/P-WS_2@CC	1.73	Ref [8]
Fe-Ni_2P@PC/Cu_xS	2.1	Ref [9]
NiCoFe-O@NF	1.7	Ref [10]
$CoMoN_x$-500 NSAs/NF	1.91	Ref [11]
NiCoP-WO_x ‖ NiFeP-WO_x	1.72	Ref [12]
Ni@NCNTs/NF-L ‖ NiFe-L	2.1	Ref [13]
N,Co-CNTs	1.9	Ref [14]
$NiFe_2O_4$@N/rGO-800	1.84	Ref [15]
NiP_2/$NiSe_2$	1.8	Ref [16]
NiSP/NF	1.85	Ref [17]
$FeN_{0.023}$/Mo_2C/C	1.72	Ref [18]
Co-P@IC/(Co-Fe)P@CC	1.67	Ref [19]
Np-NiMnFeMo	1.76	Ref [20]
Ni_2P-Fe_2P/NF	1.68	Ref [21]
NiFe(Co)P-CNT@NiCo/CP	1.92	Ref [22]
FeCo/Co_2P@NPCF	1.98	Ref [23]
O-CoP-2	1.79	Ref [24]
Co-NC/CF	1.86	Ref [25]
CoP NFs	1.92	Ref [26]

Supplementary Table 2. Thermal corrections to Gibbs free energies of different adsorbates.

Adsorbate	G (eV)[a]	$\Delta G_{0-298.15\,K}$[a]	ZPE (eV)[a]	TS (eV)[a]	ZPE (eV)[b]	TS (eV)[b]
H_2 (g)	-6.81	-0.05	0.27	0.40	0.27[b]	0.41[b]
O_2 (g)	-9.98[c]	-	-	-	0.10[b]	0.64[b]
H_2O (l)	-14.25	-0.03[c]	0.57	0.70	0.56[b]	0.67[b]
*O_lO[d]	-	0.05 ~ 0.06	0.14	0.15 ~ 0.17	0.17[b]	0.00[b]
*OH	-	0.27 ~ 0.36	0.33 ~ 0.38	0.12 ~ 0.16	0.30[b]	0.00[b]
*O	-	0.01 ~ 0.06	0.05 ~ 0.07	0.03 ~ 0.09	0.07[b]	0.00[b]
*OOH	-	0.30 ~ 0.40	0.42 ~ 0.46	0.12 ~ 0.23	0.44[e]	0.00[e]

a: This work.

b: Data cited from reference[27].

c: Calculated from equation (5) in Supplementary note 12.

d: O_l denotes the lattice oxygen atom in the LOM pathway.

e: Data cited from reference[28].

Supplementary Table 3. Correction of errors introduced by PBE functional. All units are given in eV.

	ΔG	without correction		with correction		Correction
		NiFe	MoNiFe	NiFe	MoNiFe	
LOM	ΔG_1	0.33	-0.34	0.33	-0.34	0.00
	ΔG_2	0.45	0.12	0.25	-0.08	-0.20
	ΔG_3	0.27	**0.42**[a]	0.27	**0.42**[a]	0.00
	ΔG_4	**0.75**[a]	-0.02	**0.95**[a]	0.18	0.20
	ΔG_5	-1.81	-0.19	-1.81	-0.19	0.00
AEM on Ni site	ΔG_1	-0.01	0.27	-0.01	0.27	0.00
	ΔG_2	**1.08**[a]	**1.12**[a]	**1.08**[a]	**1.12**[a]	0.00
	ΔG_3	-0.25	-0.42	-0.45	-0.62	-0.20
	ΔG_4	-0.82	-0.97	-0.62	-0.77	0.20
AEM on Fe site	ΔG_1	-0.24	-0.26	-0.24	-0.26	0.00
	ΔG_2	**1.05**[a]	**0.76**[a]	**1.05**[a]	**0.76**[a]	0.00
	ΔG_3	-0.05	0.21	-0.25	0.01	-0.20
	ΔG_4	-0.76	-0.70	-0.56	-0.50	0.20
AEM on Ni site (with oxygen vacancy)	ΔG_1	0.30	0.18	0.30	0.18	0.00
	ΔG_2	**1.14**[a]	**0.98**[a]	**1.14**[a]	**0.98**[a]	0.00
	ΔG_3	-0.64	-0.15	-0.84	-0.35	-0.20
	ΔG_4	-0.80	-1.00	-0.60	-0.80	0.20
AEM on Fe site (with oxygen vacancy)	ΔG_1	-0.29	-0.32	-0.29	-0.32	0.00
	ΔG_2	**0.87**[a]	**0.90**[a]	**0.87**[a]	**0.90**[a]	0.00
	ΔG_3	0.02	0.03	-0.18	-0.17	-0.20
	ΔG_4	-0.60	-0.61	-0.40	-0.41	0.20

a: The potential determining step (PDS)

Supplementary note 1

The OER performance of MoNiFe (oxy)hydroxide was compared with the benchmark RuO_2 and IrO_2 samples, as shown in **Supplementary Fig. 12a**. The MoNiFe (oxy)hydroxide delivered an overpotential of 242 mV at the current density of 10 mA/cm^2, which was much lower than RuO_2 (277 mV) and IrO_2 (363 mV). To reach a current density of 100 mA/cm^2, the MoNiFe (oxy)hydroxide required only an overpotential of 290 mV, while RuO_2 and IrO_2 needed 385 mV and 466 mV, respectively. To assess the intrinsic activity of the catalysts, the mass activity was obtained by normalizing the CV curves by loading mass (**Supplementary Fig. 12b**). The MoNiFe (oxy)hydroxide delivered a mass activity of 1910 A/g at the overpotential of 300 mV, which is much higher than that of 112 A/g and 5.56 A/g for RuO_2 and IrO_2, respectively (**Supplementary Fig. 12c**).

Supplementary note 2

The characterizations of MoNiFe (oxy)hydroxide after CP measurement were carried out. **Supplementary Fig. 13** shows the SEM images of MoNiFe (oxy)hydroxide after CP measurement. The morphology of the MoNiFe (oxy)hydroxide showed negligible change after CP measurement. The MoNiFe (oxy)hydroxide layer still uniformly coated on carbon cloths.

The metal contents in MoNiFe (oxy)hydroxide before and after CP measurement were determined by ICP-OES measurement (**Supplementary Fig. 14**). The changes in

the cation contents are within experimental error, suggesting that the Ni, Fe, and Mo contents in MoNiFe (oxy)hydroxide remained almost unchanged after CP measurement.

The crystal structure of MoNiFe (oxy)hydroxide after CP measurement was identified by high-resolution transmission electron microscopy (HRTEM) (**Supplementary Fig. 15**). The spacing between two adjacent lattice planes was quantified to be 0.21 nm (**Supplementary Fig. 15b**), which is assigned to the (105) plane of oxyhydroxide. Such value is slightly larger than that of the pristine MoNiFe (oxy)hydroxide (0.20 nm), which suggests the lattice expansion during CP measurement. The selected area electron diffraction (SAED) pattern of the MoNiFe (oxy)hydroxide after CP measurement shows clear diffraction rings of (105) and (110) plane for Ni-based oxyhydroxide (PDF-#06-0075) (**Supplementary Fig. 15c**). The diffraction rings for Ni-based hydroxide were not observed, indicating the complete conversion of hydroxide to oxyhydroxide during long-time CP measurement. As revealed in the SEM-EDS (**Supplementary Fig. 16**) and TEM-EDS (**Supplementary Fig. 15d**) mapping, the distribution of Mo, Ni, Fe elements in MoNiFe is uniform after CP measurement, and are the same as the pristine one.

The chemical composition of MoNiFe (oxy)hydroxide after CP measurement was identified by X-ray photoelectron spectroscopy (XPS) (**Supplementary Fig. 17**). Both the Fe 2*p* and Ni 2*p* remained unchanged after CP measurement comparing to the pristine one. In the O 1*s* XPS spectra, the peak of adsorbed H_2O on the surface increase obviously after CP measurement, while the peaks of defective O and lattice O do not

show noticeable changes. All these results above demonstrate that the structure and composition of MoNiFe (oxy)hydroxide remained almost unchanged during long-time operation under OER conditions, explaining the high stability of the catalysts.

Supplementary note 3

The pH-dependent activity on the RHE scale means that OER includes a non-concerted proton-electron transfer (nCPET) process. For the catalysts with the LOM mechanism, the OER reaction normally involves the nCPET process. In some literature, the proton and electron transfer process occurred in two separate reaction steps, in which the PDS is the step that involved proton transfer[29, 30]. For instance, Huang et al.[29] reported that the PDS of OER on $Na_xMn_3O_7$ is the chemical deprotonation step, while the electron transfer was accompanied by the O_2 desorption step. Nevertheless, there are also many previous literature which reported that although the electron and proton transfer both occurred in the same step, these two processes proceed sequentially instead of simultaneously[31-33]. Because it is difficult to identify whether proton transfer or electron transfer occurs first, the proton transfer step and the electron transfer step generally are shown together in the schematic illustration of pathways and energy diagrams. For example, Zhou et al.[32] reported a spinel oxide catalyst of $ZnFe_{0.4}Co_{1.6}O_4$ with pH-dependent OER activity, whose PDS (the formation of *OOH) includes decoupled proton-electron transfer pathways. Similarly, Zhu et al.[33] observed a pH-dependent OER activity of $Sr_3(Co_{0.8}Fe_{0.1}Nb_{0.1})_2O_{7-\delta}$ catalyst and proposed a lattice

oxygen mechanism with the PDS that includes both proton transfer and electron transfer.

In our work, we indeed considered two possible pathways (LOM-1 and LOM-2) in our DFT calculations. While both proton transfer and electron transfer occur on the deprotonation of *OOH step in the LOM-1 pathway (**Supplementary Fig. 22a**), the proton transfer occurs on the deprotonation of *OOH step and the electron transfer occurs on the O_2 desorption step in the LOM-2 pathway (**Supplementary Fig. 22b**). We found that the reaction barrier for the LOM-1 pathway is significantly lower than that for the LOM-2 pathway**,** as shown in **Supplementary Fig. 22c**. This result suggests that the LOM-1 pathway is more favorable for our cases.

Since we observed a strong PH dependence for the MoNiFe (oxy)hydroxide, we believe that although the proton and electron transfer both occurred in the PDS, they are actually transferred sequentially, i.e., the electron and proton transfer process are decoupled as shown in **Supplementary Fig. 23**. DFT method is known to be problematic dealing with charged systems, and it is challenging to assign charge to an atom during calculations. Therefore, it is difficult to verify the sequence of proton transfer and electron transfer in our PDS step.

Supplementary note 4

As shown in the **Supplementary Fig. 26**, the O 1*s* XPS spectra can be deconvoluted into three characteristic species, including the oxygen-metal bond in the lattice (lattice O) at ~530.1 eV, the unsaturated oxygen with low coordination (defective

O) at ~531.5 eV, and adsorbed water molecules on surface (adsorbed H_2O) at ~532.5 eV[34, 35]. To quantify the defective O in (oxy)hydroxide catalyst, the area ratio of defective O to the total area of O 1*s* spectra and to the lattice O were calculated. As shown in **Supplementary Fig. 26c**, the MoNiFe (oxy)hydroxide shows higher defective O content than NiFe (oxy)hydroxide, suggesting that the MoNiFe (oxy)hydroxide might with more unsaturated oxygen sites, which is consistent with the higher oxygen activity as revealed by DFT calculation.

Supplementary note 5

The Gibbs free energy diagrams of OER in the AEM pathway on NiFe and MoNiFe with oxygen vacancy are shown in **Supplementary Fig. 27**. The corresponding configurations of reaction intermediate are shown in **Supplementary Fig. 28**. We found that the Fe sites serve as active sites in the presence of oxygen vacancy for both NiFe and MoNiFe (oxy)hydroxide, which is similar to the case without oxygen vacancy. As shown in **Supplementary Fig. 27c**, the deprotonation of *OH in the AEM pathway serves as PDS for both NiFe and MoNiFe (oxy)hydroxide, with a barrier of 0.87 eV and 0.90 eV, respectively. The Gibbs free energy diagrams of OER in the LOM pathway on NiFe and MoNiFe with oxygen vacancy are shown in **Supplementary Fig. 29**. The corresponding configurations of reaction intermediate are shown in **Supplementary Fig. 30**. In the LOM pathway, the formation of gaseous O_2 and the deprotonation of *OOH act as PDSs for NiFe and MoNiFe (oxy)hydroxide,

with a barrier of 0.75 eV and 0.42 eV, respectively (**Supplementary Fig. 29b**), which is the same as the case without oxygen vacancy. These DFT results show that, after introducing oxygen vacancy on the surface, the reaction barrier in the LOM pathway is still lower than that in the AEM pathway. Therefore, the LOM pathway is still dominant for both NiFe and MoNiFe (oxy)hydroxide when surface defects were considered, and the MoNiFe (oxy)hydroxide exhibited a lower reaction barrier.

Supplementary note 6

Typically, transition metal atoms interact with bridging oxygen (μ-O) through π-donation in (oxy)hydroxide structures to form M-O-M moiety.[36, 37] The valence electronic configurations of Ni^{2+}, Fe^{3+}, Mo^{4+} and Mo^{6+} are $3d^8$ ($t_{2g}^6e_g^2$), $3d^5$ ($t_{2g}^3e_g^2$), $4d^2$ ($t_{2g}^2e_g^0$) and $4d^0$ ($t_{2g}^0e_g^0$), respectively, which are used to analyse the charge transfer of M-O-M moiety in NiFe, and MoNiFe (oxy)hydroxide.[37] For the Ni-O-Fe moiety in NiFe (oxy)hydroxide, Fe^{3+} is with half-full occupancy in the π-symmetry (t_{2g}) *d*-orbitals while Ni^{2+} is with the fully occupied t_{2g} orbitals, resulting in the partial electron transfer from Ni^{2+} to Fe^{3+} through Ni-O-Fe moiety (**Supplementary Fig. 32**). However, for the Ni-O-Mo-O-Fe moiety in MoNiFe (oxy)hydroxide, Mo^{4+} and Mo^{6+} possess lower t_{2g} occupancy than Ni^{2+} and Fe^{3+}, so that the partial electron transfer from both Ni^{2+} and Fe^{3+} to Mo^{4+} or Mo^{6+} is stronger (**Supplementary Fig. 32**), which is consistent with the both increased intensity of Ni L-edge and Fe L-edge peaks for MoNiFe (oxy)hydroxide compared to NiFe (oxy)hydroxide (**Fig. 6b,c**). Such enhanced electron

transfer in Ni-O-Mo-O-Fe moiety results in the higher valence state of Ni in MoNiFe (oxy)hydroxide (**Fig. 6d**).

Supplementary note 7

To further quantify the higher valence states of Ni after Mo doping, X-ray photoelectron spectroscopies (XPS) for NiFe and MoNiFe (oxy)hydroxide were carried out. As shown in **Fig. 6d** inset and **Supplementary Fig. 33**, the Ni 2*p* XPS spectra can be fitted by two spin-orbit doublets and two satellite peaks (denoted as Sat.). The doublet located at 856.0 and 873.6 eV is attributed to Ni $2p_{3/2}$ and $2p_{1/2}$ of Ni^{2+}, while the doublet located at a higher binding energy of 857.5 eV and 875.1 eV correspond to Ni $2p_{3/2}$ and $2p_{1/2}$ of Ni^{3+}. The Ni^{2+} and Ni^{3+} ratios were calculated based on the area of two doublets. As shown in **Fig. 6d**, MoNiFe (oxy)hydroxide shows higher Ni^{3+} contents than NiFe (oxy)hydroxide.

As shown in **Supplementary Fig. 34**, the Fe 2*p* XPS spectra consist two peaks at ~710.7 eV and ~723.7 eV, which can be attributed to Fe^{3+}.[38] After Mo doping, the Fe 2*p* spectra shift slightly to higher energy level, suggesting an increased in the Fe valence state. Such impact of Fe valence state by Mo doping is consistent with the Fe L-edge XAS results (**Fig. 6c**).

Supplementary note 8

The weak Mott-Hubbard splitting in *d*-orbitals, that is a small *U* value, allows the LHB to locate above the redox energy of the O_2/H_2O couple (**Supplementary Fig. 38**, left), resulting in a more favorable cationic redox electrochemistry to donate electrons as an anodic potential is applied. However, with the upshifted O 2*p* band relative to Fermi level and the enlarged *U* value, the LHB band can be manipulated to penetrate the O 2*p* band and locate under the redox energy of the O_2/H_2O couple (**Supplementary Fig. 38**, right). Such an electronic manipulation makes the removing of electrons from oxygen more favorable, leading to the lattice oxygen oxidation companying with the delayed cation redox. Because of the competition of electron donation from oxygen anion and metal cations redox process, the enhanced oxygen reactivity should be reflected on the delayed cationic electrochemical redox process.

Supplementary note 9

Synthesis of MoNiFe (oxy)hydroxides: The preparation of self-reconstruction MoNiFe (oxy)hydroxide was involved three steps. First, MoS_2 nanosheets were synthesized by a hydrothermal method. Ammonium molybdate tetrahydrate [$(NH_4)_6Mo_7O_{24}\cdot 4H_2O$] and thiourea ($CH_4N_2S$) were added into deionized water and stirred 1 h by a magnetic stirrer to dissolve thoroughly. Then hydrochloric acid was added to adjust the pH to be 3. The resulting solution was transferred into a Teflon-line stainless-steel autoclave, and a piece of carbon cloths was immersed in solution as substrate. The autoclave was heated to 180 ºC for 24 h and cooled to room temperature

naturally. The obtained samples were washed with deionized water several times to remove excess reactant and then dried at 60 ºC for 5 h in vacuum. Second, the MoS_2/NiFe LDH pre-catalysts were constructed by a chemical bath. MoS_2 nanosheets were activated at -1.5 V (vs. Ag/AgCl) in 1M KOH to improve their hydrophilia, and then washed by deionized water thoroughly. The resulting samples were further immersed in a mixed solution of nickel acetate and ferrous sulfate (30 mM) for 20 min to construct the MoS_2/NiFe LDH pre-catalysts. Then the pre-catalysts were washed by deionized water thoroughly to remove excess adsorbate and dried at ambient conditions. Third, the MoS_2/NiFe LDH pre-catalysts were subjected to 5 cycles of cyclic voltammetry activation in 1 M KOH solution to obtain self-reconstruction Mo doping NiFe (oxy)hydroxide through Mo leaching. The CV activation was performed at the potential range from 0 V to 0.8 V (vs. Ag/AgCl) with a scan rate of 5 mV/s. MoNi (oxy)hydroxide and MoFe (oxy)hydroxide were synthesized in the same method except for the chemical bath using pure nickel acetate and ferrous sulfate solution, respectively.

Supplementary note 10

Synthesis of NiFe (oxy)hydroxides: 10 mL 37.5 mM mixed solution contains $Ni(NiO_3)_2$ and $Fe(NiO_3)_3$ with a certain proportion, mixed with 20 mL 10 mM KNO_3 solution (containing 23% vol% formamide), and then heated up to 80 ºC with stirring for 30 min. 0.25 M KOH solution was added into the obtained solution drop by drop to adjust the pH to 10. Afterward, the solution was bubbled by nitrogen and cool down to

room temperature naturally. The resulting solution was subjected to centrifugation to collect the precipitate. The precipitate was washed with ethanol and deionized water several times and dried at 60 ºC in a vacuum for 12 h. For the synthesis of Ni (oxy)hydroxide and Fe (oxy)hydroxide, 10 mL 37.5 mM $Ni(NiO_3)_2$ or $Fe(NiO_3)_3$ solution was used as precursor, respectively.

Supplementary note 11

Electrochemical measurements: The electrochemical measurements were performed in a three-electrode system using a CHI-660E electrochemical station. 1M KOH aqueous solution was used as electrolyte, and it was bubbled by O_2 for 30 min prior to OER measurements. The catalyst-loaded carbon cloths acted as the working electrode. The reference electrode and counter electrode were a Ag/AgCl electrode prefilled with saturated KCl aqueous solution and a Pt mesh. All electrode potentials were given versus the reversible hydrogen electrode (vs. RHE) unless otherwise mentioned. Cyclic voltammetry (CV) curves were performed at 5 mV/s. Electrochemical impedance spectra (EIS) were carried out at the overpotential of 250 mV, with an amplitude of 5 mV and a frequency range from 10^6 to 10^{-2} Hz. The Tafel slope was measured by multi-potential steps method with a stepwise increase in potential with a step time of 60 s to allow the current achieve a steady state. Chronopotentiometry tests were carried out at a constant current density of 10 mA/cm^2 for 65 h to evaluate the stability of MoNiFe (oxy)hydroxide and RuO_2 for OER. For the overall water splitting, the electrolytic cells

were assembled by MoS_2/NiFe LDH|MoNiFe coupled electrodes and commercial catalysts Pt/C|RuO_2 couple. Linear sweep voltammetry (LSV) measurements were performed at 5 mV/s. Chronopotentiometry tests were carried out at a constant current density of 10 mA/cm^2 and 100 mA/cm^2 for 20 h to evaluate the stability of electrolytic cells during long-term operation for water splitting.

Supplementary note 12

Theoretical calculation: Spin-polarized DFT calculations were performed using the Vienna ab initio simulation package (VASP)[39]. The generalized gradient approximation (GGA) of the Perdue-Burke-Ernzerhof (PBE) version[40] was used to describe the exchange-correlation interactions. The projector-augmented wave (PAW)[41] method is used to model core-valence electron interactions. An energy cutoff of up to 400 eV was used to converge the energy variance below 1 meV/atom. The Gaussian smearing method was used to determine the partial occupancies of orbitals with the smearing width of 0.1 eV. A DFT-D2 correction was adopted to account for van der Waals interactions. The Brillouin zone was sampled with a 3×3×1 Monkhorst-Pack grid. The energy and force convergence criteria were 10^{-4} eV and 0.03 eV/Å, respectively. The Hubbard-U terms for Ni and Fe were included in the calculations, with the effective U value of 4.00 and 4.30 eV for Ni and Fe, respectively. The COHP of considered atomic pairs was calculated by the Lobster code.[42-45] The basis set of Koga[46, 47] was used where valence orbitals of *s*, *sp*, and *spd* were considered for H, O, and metal elements,

respectively. Good accuracy of all COHP calculations was obtained with the charge spilling lower than 1%.

1) Optimized DFT model of OER using for both AEM and LOM process:

The slab models of NiFe (oxy)hydroxide used in AEM and LOM pathway were terminated by the (001) surface (**Supplementary Fig. 41**). For the AEM pathway, the metal site should be exposed to the reactants. Thus, two vacuum spaces were inserted along (001) and (010). To eliminate the interaction between periodic slabs, the thickness of vacuum spaces in both models was more than 10 Å. In addition, part of hydrogen atoms was removed because of the oxidation atmosphere.

To find the stable configuration of Mo doping, we have built three slab models with different Mo sites (**Supplementary Fig. 42**). The relative stability of Mo replacement was determined by calculating the formation energy (ΔE_{f}), which was computed as:

$$\Delta E_{\mathrm{f}} = E_{\mathrm{slab}} - \sum N_i E_i, \quad (1)$$

where E_{slab}, E_i and N_i are total energies of the slab model, energy, and number of the i-th element, respectively. The calculated results show that the Mo atom favors replacing the Ni site and exposes to the vacuum as this model with the most negative ΔE_{f} (**Supplementary Fig. 42d**).

2) Determining the LHB and UHB band center:

The LHB was determined by the 3*d*-orbital distribution below E_F in DOS diagrams, while the UHB was determined by the unoccupied 3*d*-orbitals distribution above E_F. The center of LHB and UHB were calculated by:

$$\bar{\varepsilon}_{\mathrm{LHB}} = \frac{\int_{-\infty}^{0} n(\varepsilon)\varepsilon d\varepsilon}{\int_{-\infty}^{0} n(\varepsilon) d\varepsilon}, \quad (2)$$

and

$$\bar{\varepsilon}_{\mathrm{UHB}} = \frac{\int_{0}^{+\infty} n(\varepsilon)\varepsilon d\varepsilon}{\int_{0}^{+\infty} n(\varepsilon) d\varepsilon}, \quad (3)$$

where ε and $n(\varepsilon)$ are the energy level and number of states at this energy level, respectively.

3) Oxygen vacancy formation energy calculation:

The formation energy of oxygen vacancy (ΔG_{O_v}) was calculated with respect to the Gibbs free energy of O_2 at 298.15 K and 1.0 bar.

$$\Delta G_{\mathrm{O_v}} = \frac{1}{2} G_{\mathrm{O_2}} + G_{\mathrm{O_v}} - G_{\mathrm{surface}}, \quad (4)$$

where $G_{\mathrm{O_2}}$, $G_{\mathrm{O_v}}$, and G_{surface} are Gibbs free energies of O_2, surface with oxygen vacancy, and the clean surface, respectively. Since DFT calculations are inaccurate at describing the oxygen molecules, the Gibbs free energy of O_2 was calculated by:

$$G_{\mathrm{O_2}} = 2G_{\mathrm{H_2O}} - 2G_{\mathrm{H_2}} + 4.92\ \mathrm{eV}, \quad (5)$$

where G_{H_2O} and G_{H_2} are Gibbs free energies of H_2O and H_2, respectively. The Gibbs free energy change of H_2O → H_2 + O_2 is 4.92 eV.

4) Energy barriers of OER calculation:

The energy barriers of OER on different surfaces were calculated based on the computational hydrogen electrode (CHE) model[27], where the electrode kinetics was determined by the adsorption Gibbs free energies from DFT calculations. The Gibbs free energies of gaseous H_2 and liquid H_2O were corrected at 298.15 K, 1.0 bar and 298.15 K, 0.035 bar from vibrational frequency calculations. The Gibbs free energy of gaseous O_2 was obtained from equation (5). The results in **Supplementary Table 2** show good agreement with references[27, 28].

For the AEM pathway in an alkaline electrolyte, the four-electron reactions are:

$$* + OH^- \rightarrow *OH + e^-, \quad (6)$$

$$*OH + OH^- \rightarrow *O + H_2O\ (l) + e^-, \quad (7)$$

$$*O + OH^- \rightarrow *OOH + e^-, \quad (8)$$

$$*OOH + OH^- \rightarrow * + O_2\ (g) + H_2O\ (l) + e^-, \quad (9)$$

where "*" represents the adsorption sites, which are generally the exposed metal sites.

The configurations of AEM pathway are shown in **Supplementary Fig. 43**. The free energy changes of each step can be calculated as:

$$\Delta G_1 = G(*OH) + 0.5\ G(H_2) - G(*) - G(H_2O) - eU, \quad (10)$$

$$\Delta G_2 = G(*O) + 0.5\ G(H_2) - G(*OH) - eU, \quad (11)$$

$$\Delta G_3 = G(*OOH) + 0.5\ G(H_2) - G(*O) - G(H_2O) - eU, \quad (12)$$

$$\Delta G_4 = G(*) + G(O_2) + 0.5\ G(H_2) - G(*OOH) - eU, \quad (13)$$

where U is the potential with respect to the normal hydrogen electrode (NHE).

The calculated overpotential (η) was then determined by:

$$\eta = \mathrm{Max}\{\Delta G_1, \Delta G_2, \Delta G_3, \Delta G_4\} \quad (14)$$

The LOM pathway includes five steps, which are:

$$*O_lH + OH^- \rightarrow *O_l + H_2O\ (l) + e^-, \quad (15)$$

$$*O_l + OH^- \rightarrow *O_lOH + e^-, \quad (16)$$

$$*O_lOH + OH^- \rightarrow *O_lO + H_2O + e^-, \quad (17)$$

$$*O_lO \rightarrow * + O_2, \quad (18)$$

$$* + OH^- \rightarrow *O_lH + e^-, \quad (19)$$

where "*" represents the vacancy sites. O_l denotes the lattice oxygen atoms.

The configurations of LOM pathway are shown in **Supplementary Fig. 44**. The energy barriers of LOM pathway were calculated by:

$$\Delta G_1 = G(*O_l) + 0.5\ G(H_2) - G(*O_lH) - eU, \quad (20)$$

$$\Delta G_2 = G(*O_lOH) + 0.5\ G(H_2) - G(H_2O) - G(*O_l) - eU, \quad (21)$$

$$\Delta G_3 = G(*O_lO) + 0.5\ G(H_2) - G(*O_lOH) - \mathrm{e}U, \quad (22)$$

$$\Delta G_4 = G(*) + G(O_2) - G(*O_lO), \quad (23)$$

$$\Delta G_5 = G(*O_lH) + 0.5\ G(H_2) - G(H_2O) - G(*), \quad (24)$$

The overpotential of LOM is calculated by:

$$\eta = \mathrm{Max}\{\Delta G_1, \Delta G_2, \Delta G_3, \Delta G_4, \Delta G_5\} \quad (25)$$

The systematic errors of the PBE functional were considered, which arise from the difficulty in describing the triplet ground state of the O-O bond using DFT[48]. Compared with the experimental results, the PBE functional used in this work may overestimate the total energy of O-O bond by 0.20 eV with a standard deviation of 0.03 eV[48]. As shown in **Supplementary Table 3**, the systematic errors caused by PBE functional will

not affect our conclusions in this work. Specifically, the correction of PBE functional will lower the total energy of OO* and OOH* by 0.2 eV. Therefore, for those sites where the peroxide species are not involved in the PDS steps, the overpotential value remains unchanged. On the other hand, the general trends still hold if peroxide species are involved in the PDS. For example, the overpotential of OER on the surface of NiFe (oxy)hydroxide increases by 0.2 eV after the correction. In contrast, the overpotential on MoNiFe surfaces remains the same because the energies of OO* and OOH* are shifted with the same magnitude.

References

1. Cao, D., Xu, H., Cheng, D. Branch-leaf-shaped CuNi@NiFeCu nanodendrites as highly efficient electrocatalysts for overall water splitting. *Appl. Catal. B* **298**, 120600 (2021).
2. Oh, N. K., *et al.* Highly efficient and robust noble-metal free bifunctional water electrolysis catalyst achieved via complementary charge transfer. *Nat. Commun.* **12**, 4606 (2021).
3. Chen, M., *et al.* Remarkable synergistic effect in cobalt-iron nitride/alloy nanosheets for robust electrochemical water splitting. *J. Energy Chem.* **65**, 405-414 (2022).
4. Zeng, Y., *et al.* Construction of hydroxide pn junction for water splitting electrocatalysis. *Appl. Catal. B* **292**, 120160 (2021).
5. Gultom, N. S., Abdullah, H., Hsu, C.-N., Kuo, D.-H. Activating nickel iron layer double hydroxide for alkaline hydrogen evolution reaction and overall water splitting by electrodepositing nickel hydroxide. *Chem. Eng. J.* **419**, 129608 (2021).
6. Zhang, Y., *et al.* Modulating carbon-supported transition metal oxide by electron-giving and electron-absorbing functional groups towards efficient overall water splitting. *Chem. Eng. J.* **416**, 129124 (2021).
7. Dai, K., Zhang, N., Zhang, L., Yin, L., Zhao, Y., Zhang, B. Self-supported Co/CoO anchored on N-doped carbon composite as bifunctional electrocatalyst for efficient overall water splitting. *Chem. Eng. J.* **414**, 128804 (2021).
8. Paudel, D. R., Pan, U. N., Singh, T. I., Gudal, C. C., Kim, N. H., Lee, J. H. Fe and P doped 1T-phase enriched $WS_2$3D-dendritic nanostructures for efficient overall water splitting. *Appl. Catal. B* **286**, 119897 (2021).
9. Tran, D. T., Le, H. T., Hoa, V. H., Kim, N. H., Lee, J. H. Dual-coupling ultrasmall iron-Ni_2P into P-doped porous carbon sheets assembled Cu_xS nanobrush arrays for overall water splitting. *Nano Energy* **84**, 105861 (2021).
10. Wang, Z., *et al.* Contemporaneous inverse manipulation of the valence configuration to preferred Co^{2+} and Ni^{3+} for enhanced overall water electrocatalysis. *Appl. Catal. B* **284**, 119725 (2021).
11. Lu, Y., *et al.* Bimetallic Co-Mo nitride nanosheet arrays as high-performance bifunctional electrocatalysts for overall water splitting. *Chem. Eng. J.* **411**, 128433 (2021).
12. Kim, D., Jeong, Y., Roh, H., Lim, C., Yong, K. Biomimetic 2D-Ni(Co,Fe)P/1D-WO_x nanocoral reef electrocatalysts for efficient water splitting. *J. Mater. Chem. A* **9**, 10909-10920 (2021).
13. Yuan, H., *et al.* Laser patterned and bifunctional Ni@N-doped carbon nanotubes as electrocatalyst and photothermal conversion layer for water splitting driven by thermoelectric device. *Appl. Catal. B* **283**, 119647 (2021).
14. Jin, Q., Ren, B., Cui, H., Wang, C. Nitrogen and cobalt co-doped carbon nanotube films as binder-free trifunctional electrode for flexible zinc-air battery and self-powered overall water splitting. *Appl. Catal. B* **283**, 119643 (2021).
15. Cao, L., Li, Z., Su, K., Zhang, M., Cheng, B. Rational design of hollow oxygen deficiency-enriched $NiFe_2O_4$@N/rGO as bifunctional electrocatalysts for overall water splitting. *J. Energy Chem.* **54**, 595-603 (2021).
16. Yang, L., Huang, L., Yao, Y., Jiao, L. In-situ construction of lattice-matching $NiP_2/NiSe_2$

heterointerfaces with electron redistribution for boosting overall water splitting. *Appl. Catal. B* **282**, 119584 (2021).

17. Marquez-Montes, R. A.*, et al.* Mass transport-enhanced electrodeposition of Ni-S-P-O films on nickel foam for electrochemical water splitting. *J. Mater. Chem. A* **9**, 7736-7749 (2021).
18. Han, N., Luo, S., Deng, C., Zhu, S., Xu, Q., Min, Y. Defect-rich $FeN_{0.023}/Mo_2C$ heterostructure as a highly efficient bifunctional catalyst for overall water-splitting. *ACS Appl. Mater. Interfaces* **13**, 8306-8314 (2021).
19. Zhu, Y.*, et al.* Double functionalization strategy toward Co-Fe-P hollow nanocubes for highly efficient overall water splitting with ultra-low cell voltage. *Chem. Eng. J.* **405**, 127002 (2021).
20. Liu, H.*, et al.* Free-standing nanoporous NiMnFeMo alloy: An efficient non-precious metal electrocatalyst for water splitting. *Chem. Eng. J.* **404**, 126530 (2021).
21. Wu, L.*, et al.* Heterogeneous bimetallic phosphide Ni_2P-Fe_2P as an efficient bifunctional catalyst for water/seawater splitting. *Adv. Funct. Mater.* **31**, 2006484 (2020).
22. Wang, Z., Wei, C., Zhu, X., Wang, X., He, J., Zhao, Y. A hierarchical carbon nanotube forest supported metal phosphide electrode for efficient overall water splitting. *J. Mater. Chem. A* **9**, 1150-1158 (2021).
23. Shi, Q.*, et al.* High-performance trifunctional electrocatalysts based on $FeCo/Co_2P$ hybrid nanoparticles for zinc-air battery and self-powered overall water splitting. *Adv. Energy Mater.* **10**, 1903854 (2020).
24. Zhou, G.*, et al.* Regulating the electronic structure of CoP nanosheets by O incorporation for high-efficiency electrochemical overall water splitting. *Adv. Funct. Mater.* **30**, 1905252 (2019).
25. Huang, H.*, et al.* Rapid and energy-efficient microwave pyrolysis for high-yield production of highly-active bifunctional electrocatalysts for water splitting. *Energy Environ. Sci.* **13**, 545-553 (2020).
26. Ji, L., Wang, J., Teng, X., Meyer, T. J., Chen, Z. CoP Nanoframes as Bifunctional Electrocatalysts for Efficient Overall Water Splitting. *ACS Catalysis* **10**, 412-419 (2019).
27. J. K. Nørskov, J. R., A. Logadottir, L. Lindqvist. Origin of the overpotential for oxygen reduction at a fuel-cell cathode. *J. Phys. Chem. B* **108**, 17886-17892 (2004).
28. Bajdich, M., Garcia-Mota, M., Vojvodic, A., Norskov, J. K., Bell, A. T. Theoretical investigation of the activity of cobalt oxides for the electrochemical oxidation of water. *J. Am. Chem. Soc.* **135**, 13521-13530 (2013).
29. Huang, Z.-F.*, et al.* Tuning of lattice oxygen reactivity and scaling relation to construct better oxygen evolution electrocatalyst. *Nat. Commun.* **12**, 3992 (2021).
30. Pan, Y.*, et al.* Direct evidence of boosted oxygen evolution over perovskite by enhanced lattice oxygen participation. *Nat Commun* **11**, 2002 (2020).
31. Giordano, L.*, et al.* pH dependence of OER activity of oxides: Current and future perspectives. *Catalysis Today* **262**, 2-10 (2016).
32. Zhou, Y.*, et al.* Enlarged CoO Covalency in Octahedral Sites Leading to Highly Efficient Spinel Oxides for Oxygen Evolution Reaction. *Adv Mater* **30**, e1802912 (2018).
33. Zhu, Y.*, et al.* Boosting oxygen evolution reaction by activation of lattice-oxygen sites in layered Ruddlesden-Popper oxide. *EcoMat* **2**, 12021 (2020).
34. Sun, H.*, et al.* Rh-engineered ultrathin NiFe-LDH nanosheets enable highly-efficient overall

water splitting and urea electrolysis. *Appl. Catal. B: Environ.* **284**, 119740 (2021).

35. Asnavandi, M., Yin, Y., Li, Y., Sun, C., Zhao, C. Promoting oxygen evolution reactions through introduction of oxygen vacancies to benchmark NiFe-OOH catalysts. *ACS Energy Lett.* **3**, 1515-1520 (2018).
36. Zhang, N.*, et al.* Lattice oxygen activation enabled by high-valence metal sites for enhanced water oxidation. *Nat. Commun.* **11**, 4066 (2020).
37. Jiang, J.*, et al.* Atomic-level insight into super-efficient electrocatalytic oxygen evolution on iron and vanadium co-doped nickel (oxy)hydroxide. *Nat. Commun.* **9**, 2885 (2018).
38. Liang, C.*, et al.* Exceptional performance of hierarchical Ni-Fe oxyhydroxide@NiFe alloy nanowire array electrocatalysts for large current density water splitting. *Energy Environ. Sci.* **13**, 86-95 (2020).
39. Kresse, G., Furthmuller, J. Efficiency of ab-initio total energy calculations for metals and semiconductors using a plane-wave basis set. *Comput. Mater. Sci.* **6**, 15-50 (1996).
40. Perdew, J. P., Burke, K., Ernzerhof, M. Generalized gradient approximation made simple. *Phys. Rev. Lett.* **77**, 3865-3868 (1996).
41. Blochl, P. E. Projector augmented-wave method. *Phys. Rev. B Condens. Matter.* **50**, 17953-17979 (1994).
42. Dronskowski, R., Blochl, P. E. Crystal orbital Hamilton populations (COHP). Energy-resolved visualization of chemical bonding in solids based on density-functional calculations. *J. Phys. Chem.* **97**, 8617-8624 (1993).
43. Maintz, S., Deringer, V. L., Tchougreeff, A. L., Dronskowski, R. Analytic projection from plane-wave and PAW wavefunctions and application to chemical-bonding analysis in solids. *J. Comput. Chem.* **34**, 2557-2567 (2013).
44. Maintz, S., Deringer, V. L., Tchougreeff, A. L., Dronskowski, R. LOBSTER: A tool to extract chemical bonding from plane-wave based DFT. *J. Comput. Chem.* **37**, 1030-1035 (2016).
45. Deringer, V. L., Tchougreeff, A. L., Dronskowski, R. Crystal orbital Hamilton population (COHP) analysis as projected from plane-wave basis sets. *J. Phys. Chem. A* **115**, 5461-5466 (2011).
46. Koga, T., Kanayama, K., Watanabe, T., Imai, T., Thakkar, A. J. Analytical Hartree-Fock wave functions for the atoms Cs to Lr. *Theor. Chem. Acc.* **104**, 411-413 (2000).
47. Li, T.*, et al.* Anchoring $CoFe_2O_4$ Nanoparticles on N-Doped Carbon Nanofibers for High-Performance Oxygen Evolution Reaction. *Adv Sci (Weinh)* **4**, 1700226 (2017).
48. Christensen, R., Hansen, H. A., Dickens, C. F., Nørskov, J. K., Vegge, T. Functional Independent Scaling Relation for ORR/OER Catalysts. *The Journal of Physical Chemistry C* **120**, 24910-24916 (2016).
